# Wilson Loop as a Tool to Investigate Chirality-Induced Spin Selectivity: Role of Vibrations and Multiple Channels

**DOI:** 10.1021/acs.jpclett.6c00585

**Published:** 2026-05-09

**Authors:** L. Celada, D. K. A. Phan Huu, A. Chiesa, P. Santini, L. Griguolo, S. Carretta

**Affiliations:** † Dipartimento di Scienze Matematiche, Fisiche e Informatiche, 9370 Università di Parma, Parco Area delle Scienze, 53/A, I-43124 Parma, Italy; ‡ Gruppo Collegato di Parma, INFN-Sezione Milano-Bicocca, I-43124 Parma, Italy; § UdR Parma, INSTM, I-43124 Parma, Italy

## Abstract

Chirality-induced spin selectivity (CISS), whereby electrons traveling through chiral molecules become spin-polarized, is an increasingly active yet still poorly understood phenomenon. Simple theoretical tools to identify the conditions for spin polarization are, therefore, highly desirable. Here, we show that the Wilson loop provides a compact and transparent criterion for the emergence of spin polarization in electrons transmitted through chiral chains. We illustrate our approach using single-electron tight-binding models relevant to many organic molecules displaying CISS. Besides reproducing known results on the need for multiple transport channels in purely electronic models, the Wilson loop allows us to study the different roles of Holstein and Peierls coupling of electrons to vibrations, finding that only the latter enable spin polarization even in single-channel models. This formulation provides a path-based picture of spin-dependent interference between electronic and vibronic pathways and can be readily extended to arbitrary numbers of electronic sites and vibrational modes.

## Graphical abstract

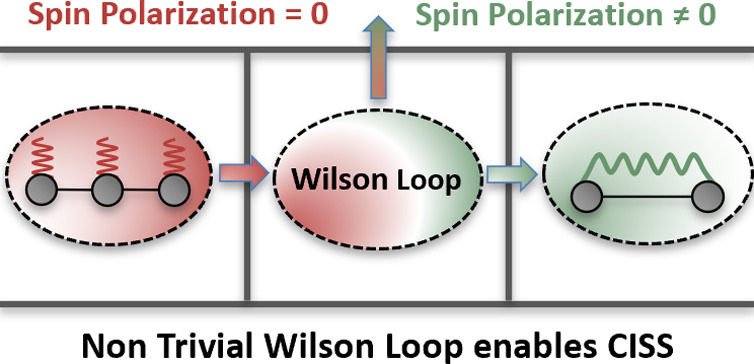

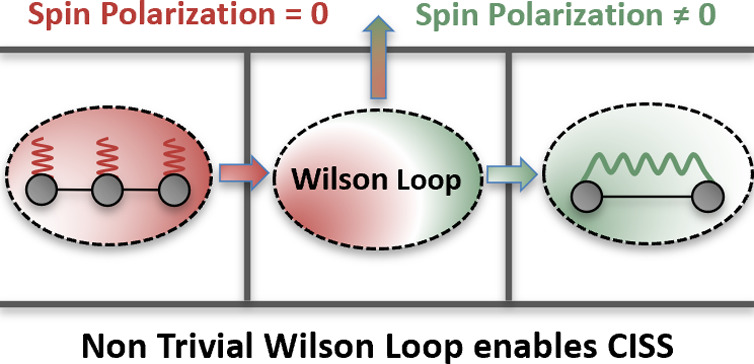

Chirality-induced spin selectivity (CISS) refers to a spin-dependent transmission of electrons passing through chiral molecules or material, even in the absence of external magnetic fields.^1,2^ Over the past decade, numerous experiments have reported sizable spin polarization in photoemission^3,4^ and spin-dependent current in electron transport through organic molecules,^5^ DNA,^6–9^ and chiral crystals.^10^ More recently, spin selectivity has also been observed in electron-transfer processes, where growing evidence points to similar trends.^11–13^ These observations have stimulated considerable interest in potential applications of CISS.^14–16^ At the same time, the microscopic origin of the effect remains an open question.^17–20^ Many theoretical approaches have been proposed to explain CISS.^21–33^ These include models based solely on spin–orbit coupling (SOC)^34–36^ or including electron–electron interactions,^37,38^ and electron–vibration couplings.^39–43^ Despite this diversity of approaches, a unified framework identifying the necessary conditions for the emergence of spin polarization in electrons transmitted through chiral molecules is still missing. While realistic experimental setups for CISS involve open systems, where molecules are coupled to the environment and/or to electrodes and additional interfacial effects may arise, it is still highly desirable to identify minimal microscopic ingredients that enable spin polarization. In this spirit, we focus here on coherent model Hamiltonians and seek a simple theoretical tool that can be applied across different physical scenarios.

Here we investigate the CISS effect by means of the Wilson loop (WL), a theoretical method widely used in high-energy and condensed-matter physics to characterize gauge fields and topological properties, encoding the phase accumulated by a particle moving along a closed path.^44–46^ Specifically, we show that the WL analysis easily provides insight into the necessary conditions for spin polarization for model Hamiltonians describing CISS.

First, we address the fundamentals of WL analysis, defining *trivial* and *nontrivial* WLs and their relations to spin polarization. Second, we apply WL analysis to purely electronic open tight-binding Hamiltonians with SOC, which are known to provide a good description of several organic molecules where CISS was observed,^11,47^ including DNA-based systems.^34,48^ In this context, WL analysis recovers known results on single- and multiple-channel tight-binding models (Figure 1a,d). In particular, we show that the trivial WL condition is equivalent to the identification of gauge transformation which eliminates the spin dependence from the Hamiltonian. Third, we include the coupling of vibrations with electronic degrees of freedom in single-channel models, and we demonstrate that the WL allows us to distinguish the role of Holstein and Peierls modes (Figure 1b,c). Although previous numerical studies have demonstrated that Peierls modes induce spin polarization,^29,47,49^ we provide here, to the best of our knowledge, the first formal theoretical interpretation.

**Figure 1:**
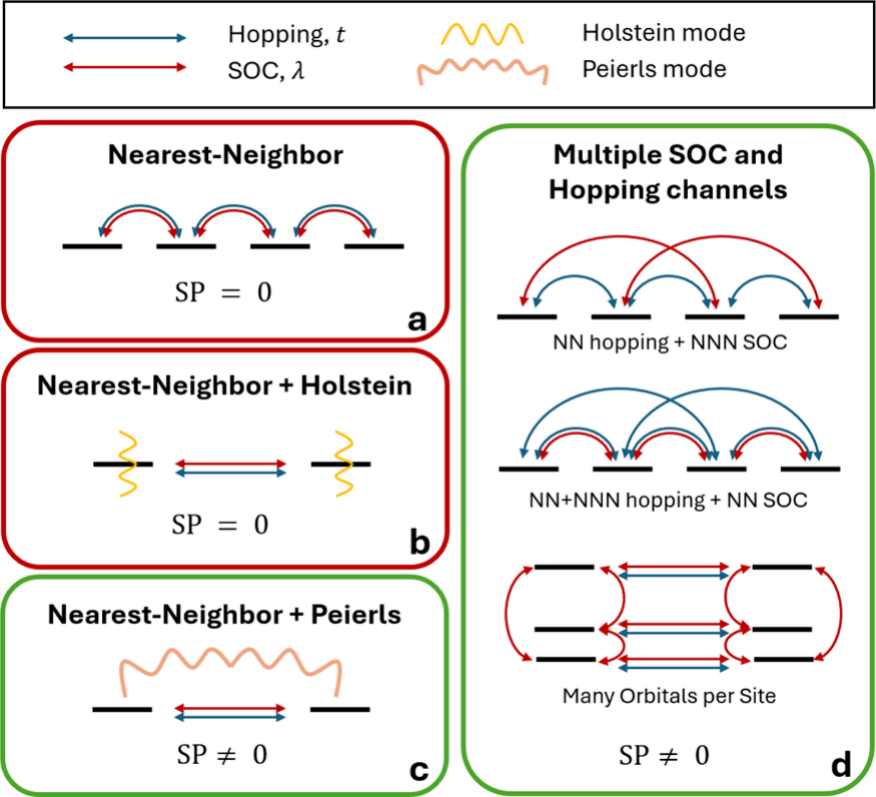
Schematic representation of the tight-binding models discussed in this work. Red outlines denote models where a local spin rotation removes all spin dependence and no spin polarization emerges. Green outlines denote models where this mapping fails, because of Peierls coupling or multiple hopping/SOC channels, resulting in finite spin polarization. The symbols indicate hopping, SOC, and vibronic modulations included in each case.

These results show the power of the WL to establish necessary conditions for spin polarization and its connection to the *presence* or *breaking* of a site-local spin gauge symmetry: whenever such a symmetry exists, the Hamiltonian is equivalent to a spinless model and CISS is forbidden; when it is broken (by Peierls coupling or by multiple transport channels), transient spin polarization becomes possible. Compared to the gauge transformation, the WL provides a clear picture based on interference paths and is easily extended to an arbitrary number of electronic sites and vibrational modes.

### Wilson Loop Analysis

The WL is a quantity that encodes the net transformation acquired by an internal degree of freedom when a particle moves along a closed path. In the present context, this internal degree of freedom is the electron spin, and the WL describes the total spin rotation accumulated as an electron propagates through a sequence of spin-dependent hopping processes and returns to its initial position.

In tight-binding models with SOC, electron propagation between neighboring sites is described by matrices acting in spin space. These matrices can be interpreted as local spin rotations associated with each bond of the lattice. When an electron traverses a closed path, the ordered product of these bond-dependent spin rotations defines the WL. More precisely, given a tight-binding Hamiltonian with spin-dependent hopping operators, one can associate to each oriented bond (*i* → *j*) an SU(2) matrix *U*_*ij*_. This amounts to a projection of a spin-dependent hopping operator on the spin degree of freedom. For any closed oriented path C = {*i*_1_ → *i*_2_ → ··· → *i*_*n*_ → *i*_1_} in the lattice, the corresponding WL, W(C) , is defined as the ordered product

1 $$W\left(C\right)\equiv U_{i_{1}i_{2}}U_{i_{2}i_{3}}\cdot \cdot \cdot U_{i_{n}i_{1}}$$

where the order reflects the sequence of bonds along the path. The WL W(C) ∈ SU(2) is invariant under arbitrary site-dependent spin rotations and therefore provides a characterization of the spin evolution. The WL is defined *trivial* when it is proportional to the identity, and *nontrivial* in all other cases.

Although infinitely many closed paths can be constructed in principle, not all of the WLs are independent. They can all be generated from an elementary set of loops^45^ depending on the lattice connectivity, such as a single loop in a ring or triangular plaquettes in multichannel models (see below). The criterion is then simple: spin polarization is forbidden if all elementary loops are trivial, while it is symmetry-allowed if at least one elementary WL is nontrivial. This condition is equivalent to finding a site-local spin gauge transformation (i.e., a set of independent SU(2) rotations of the spin states at each lattice site) which makes the model effectively spin-independent. As a result, polarization is not allowed, but the electron spin state still undergoes coherent rotations along its trajectory, as discussed in the literature on spin coherent evolution in electron transfer reactions and chiral systems.^36,50,51^ Conversely, the site-local spin gauge is broken in the presence of nontrivial elementary WLs.

The physical relevance of the WL becomes evident when quantum propagation is viewed as a superposition of all paths an electron may follow.^52^ Each path contributes an amplitude, and each step along the path can induce spin rotation. When two distinct paths connect the same points, their amplitudes interfere and any difference in the accumulated spin rotation produces a spin-dependent phase. As demonstrated in the Supporting Information, the WL provides a compact measure of this relative spin phase for any closed set of paths in the Hamiltonian and hence a general criterion for identifying spin-dependent interference phenomena, which can be applied both to steady-state transport and to real-time electron-transfer dynamics.

### Purely Electronic Tight-Binding Models

In this section, we adopt the WL criterion in the simplest setting of purely electronic single- and multiple-channel tight-binding models, which serve as a benchmark for more complex situations discussed later.

### (1) Single Channel

We first consider an open tight-binding chain of *N* sites where both hopping and SOC couple the same pairs of neighboring sites (Figure 1a):

2 $$H_{0}=\overset{N}{\underset{j=1}{\sum }}\underset{\sigma }{\sum }\epsilon_{j}c_{j\sigma }^{†}c_{j\sigma }-\overset{N-1}{\underset{j=1}{\sum }}\underset{\sigma }{\sum }\left[t_{j}c_{j\sigma }^{†}c_{j+1\sigma }-i\lambda_{j}c_{j\sigma }^{†}{\mathrm{v⃗}}_{j}\cdot \mathrm{\sigma ⃗}c_{j+1\sigma }+\mathrm{h.c.}\right]$$

where $c_{j\sigma }^{†}$ (*c*_*jσ*_) creates (annihilates) an electron at site *j* with spin σ, *t*_*j*_ denotes the spin-independent hopping amplitude, and $\lambda_{j}{\mathrm{v⃗}}_{j}\cdot \mathrm{\sigma ⃗}$ represents the SOC acting on bond (*j*, *j* + 1). This specific form of the SOC term is related to the chirality of the system through the form of ${\mathrm{v⃗}}_{j}$ , as explained in the Supporting Information.

The key observation is that, in a single-channel chain, the spin-dependent hopping matrix on each bond can always be written as a single SU(2) rotation multiplied by a real amplitude,

3 $$T_{j}\equiv -t_{j}1+i\lambda_{j}{\mathrm{v⃗}}_{j}\cdot \mathrm{\sigma ⃗}=-R_{j}U_{j}$$

with $U_{j}=e^{-i\phi_{j}{\mathrm{n̂}}_{j}\cdot \mathrm{\sigma ⃗}}$ , where ${\mathrm{n̂}}_{j}={\mathrm{v⃗}}_{j}/\left|{\mathrm{v⃗}}_{j}\right|$ denotes the unit vector specifying the axis of the spin rotation induced by the SOC on bond (*j*, *j* + 1) and

4 $$R_{j}=\sqrt{{t_{j}}^{2}+{\Lambda_{j}}^{2}},,\Lambda_{j}=\lambda_{j}∥{\mathrm{v⃗}}_{j}∥,,\phi_{j}=\arctan\left(\Lambda_{j}/t_{j}\right)$$

As shown in the Supporting Information, this implies that the spin dynamics along the chain consist of a sequence of local SU(2) rotations. In an open chain, closed paths can be formed only by traversing the same bonds in opposite directions. As a result, the ordered product of the corresponding SU(2) rotations reduces to the identity, leading to a trivial WL. Therefore, for any closed path C in an open chain one finds

5 $$W\left(C\right)=\underset{\left(j\to j+1\right)\in C}{\prod }U_{j}=1$$

proving that all elementary WLs are trivial. In fact, all spin-dependent effects can be removed by an explicit site-dependent spin rotation, which maps the Hamiltonian onto a spin-independent form. Introducing the cumulative rotation $W_{j+1}={U_{j}}^{-1}W_{j}$ and defining $d_{j,\sigma }=W_{j}^{†}c_{j,\sigma }$ , the Hamiltonian is mapped onto

6 $$\mathrm{H̃}=\overset{N}{\underset{j=1}{\sum }}\underset{\sigma }{\sum }\epsilon_{j}d_{j,\sigma }^{†}d_{j,\sigma }-\overset{N-1}{\underset{j=1}{\sum }}R_{j}\underset{\sigma }{\sum }\left(d_{j,\sigma }^{†}d_{j+1,\sigma }+h.c.\right)$$

which is fully spin independent. Therefore, single-channel open chains cannot support spin polarization.

For completeness, we briefly comment on the case of closed single channel tight binding models, realized, e.g., when periodic boundary conditions are imposed. Closing the chain into a ring introduces a WL associated with one full traversal of the system. This WL is nontrivial aside for very specific *t*/λ ratios (see Supporting Information); thus periodic boundary conditions may lead to spin polarization. Indeed, this WL analysis directly connects to the literature on equilibrium persistent spin currents in systems with periodic boundary conditions and Rashba SOC terms.^53^

### (2) Multiple Channels

We now turn to tight-binding models in which electron transport can occur through multiple channels, which typically arises in molecular systems when hopping and/or SOC are not restricted to nearest neighbors.^35,37^ Another important realization is provided by systems where charge transport involves more than one orbital per site.^54^ In this case, nontrivial WLs associated with elementary closed paths of the lattice appear. We show this by considering as an example an open tight-binding chain with nearest-neighbor (NN) hopping and next-nearest-neighbor (NNN) SOC (Figure 1d, top):

7 $$H=\overset{N}{\underset{j=1}{\sum }}\underset{\sigma }{\sum }\epsilon_{j}c_{j\sigma }^{†}c_{j\sigma }-\overset{N-1}{\underset{j=1}{\sum }}\underset{\sigma }{\sum }t_{j}c_{j\sigma }^{†}c_{j+1\sigma }+i\overset{N-2}{\underset{j=1}{\sum }}\underset{\sigma }{\sum }\lambda_{j}c_{j\sigma }^{†}\left({\mathrm{v⃗}}_{j}\cdot \mathrm{\sigma ⃗}\right)c_{j+2\sigma }+\mathrm{h.c.}$$

In contrast to the single-channel case, when spin-independent and spin-dependent terms act on bonds of different spatial range, the lattice contains elementary closed paths. Each site *j* is associated with a triangular plaquette, △, connecting sites *j*, *j* + 1, and *j* + 2. The ordered product of the SU(2) link matrices around such a plaquette defines a local WL,

8 $$W_{j}^{△}=U_{j,j+1}U_{j+1,j+2}{\left(U_{j,j+2}\right)}^{-1}$$

which encodes the relative spin rotation accumulated along the two inequivalent paths connecting *j* and *j* + 2. In the simplest uniform case with NN hopping *t* and NNN SOC λv⃗·σ⃗ , the situation is schematically illustrated in Figure 2. One finds

9 $$W_{j}^{△}=e^{i\left(\pi /2\right)\mathrm{n̂}\cdot \mathrm{\sigma ⃗}},,\mathrm{Tr}\;W_{j}^{△}=2\cos\left(\frac{\pi }{2}\right)=0$$

indicating a nontrivial local SU(2) phase. Physically, this means that an electron propagating from site *j* to *j* + 2 can follow two inequivalent paths, either sequential NN hoppings or a direct NNN spin–orbit process, which accumulate a relative SU(2) phase corresponding to $W_{j}^{△}$ . The interference between these two paths is therefore controlled by the trace of this local WL, which quantifies the relative SU(2) rotation accumulated along the two trajectories. In the present case, $Tr\;W_{j}^{△}$ = 0 implies that the two paths generate orthogonal spin rotations, so that their spin-averaged interference term vanishes. Physically, this reflects a maximal mismatch between the spin orientations produced by the NN+NN and NNN processes. Equivalently, the nontriviality of $W_{j}^{△}$ provides a gauge-invariant signature that the two paths cannot be made equivalent by any site-local SU(2) transformation. Indeed, since

10 $$W_{j}^{△}\neq 1$$

no site-local spin rotation can simultaneously align the spin dynamics on all bonds. This intrinsic SU(2) mismatch between inequivalent paths is precisely what allows spin-dependent interference and, consequently, spin polarization in multiple-channel tight-binding models.

**Figure 2:**
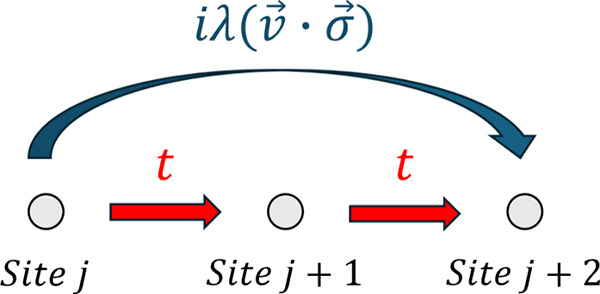
Schematic representation of the two interfering paths connecting sites *j* and *j* + 2 in the multiple transport channels model with nearest-neighbor hopping *t* (red arrows) and next-nearest-neighbor spin–orbit coupling iλ(v⃗·σ⃗) (blue arc).

A natural extension of the previous models is to consider multiple orbitals per site (Figure 1d, bottom). A convenient way to analyze this situation is to “unfold” the multiorbital structure into an effective linear chain, where each orbital label is treated as an additional site index. Yet, all the considerations of the previous sections apply and multiorbital systems do not introduce qualitatively new mechanisms for CISS: they realize a concrete instance of the multiple transport channels scenario discussed above.

### Electron-Vibration Coupling: Holstein Modes

We now consider the coupling of electrons to Holstein-type vibrational modes, which modulate the on-site (orbital) energies. Examples of these modes are local vibrations associated with individual molecular fragments, such as in-plane stretchings of π-conjugated moieties in charge-transfer triads, or hydrogen bond stretchings between base pairs in DNA.^11,13^

The Hamiltonian with a single Holstein mode becomes

11 $$H_{Hol}=H_{0}+\hbar \omega_{0}\left(a^{†}a+\frac{1}{2}\right)+\overset{N}{\underset{j=1}{\sum }}g_{j}\left(a^{†}+a\right)\underset{\sigma }{\sum }c_{j\sigma }^{†}c_{j\sigma }$$

where *ℏω*_0_ is the vibrational frequency, *a* ^†^ (*a*) creates (annihilates) a vibrational quantum, and *g*_*j*_ denotes the electron–vibration coupling strength at the *j*-th site.

It is useful to rewrite the Hamiltonian by means of the Lang–Firsov unitary transformation ${\mathrm{H̃}}_{Hol}=e^{S}H_{Hol}e^{-S}$ with

12 $$S=\overset{N}{\underset{j=1}{\sum }}\frac{g_{j}}{\hbar \omega_{0}}\left(a^{†}-a\right)\underset{\sigma }{\sum }c_{j\sigma }^{†}c_{j\sigma }$$

This yields (see Supporting Information for details):

$${\mathrm{H̃}}_{Hol}=\overset{N}{\underset{j=1}{\sum }}\underset{\sigma }{\sum }\left(\epsilon_{j}-\frac{{g_{j}}^{2}}{\hbar \omega_{0}}\right)c_{j\sigma }^{†}c_{j\sigma }+\hbar \omega_{0}\left(a^{†}a+\frac{1}{2}\right)-\overset{N-1}{\underset{j=1}{\sum }}\underset{\sigma }{\sum }\left[t_{j}c_{j\sigma }^{†}c_{j+1\sigma }\right]e^{\left(g_{j}-g_{j+1}\right)/\hbar \omega_{0}\left(a^{†}-a\right)}+\mathrm{h.c.}+\overset{N-1}{\underset{j=1}{\sum }}\underset{\sigma }{\sum }\left[i\lambda_{j}c_{j\sigma }^{†}{\mathrm{v⃗}}_{j}\cdot \mathrm{\sigma ⃗}c_{j+1\sigma }\right]e^{\left(g_{j}-g_{j+1}\right)/\hbar \omega_{0}\left(a^{†}-a\right)}+\mathrm{h.c.}$$ 13

where the electron-vibration coupling has been absorbed into a renormalization of the on-site energies and an “exponential dressing” of the hopping and SOC operators.

Leaving aside the trivial case of *g*_*j*_ = *g*_*j*+1_, accounting for Holstein modes amounts to a renormalization of the NN hopping and SOC amplitudes. Hence, the spin-dependent and spin-independent parts still act on the same bonds and can be eliminated by suitable site-local spin rotation. In WL terms, all closed paths remain trivial, and the SU(2) spin structure remains unchanged. The same conclusion holds in the presence of multiple independent Holstein modes (see Supporting Information). Each mode contributes additively to the Lang–Firsov displacement, but the Fermionic spin structure is left unchanged. Consequently, the WL remains trivial, and the site-local spin gauge symmetry is preserved. We therefore conclude that Holstein vibrations can strongly affect the charge dynamics but cannot induce spin polarization in open single-channel tight binding models.

### Electron-Vibration Coupling: Peierls Modes

We now turn to Peierls-type vibrational couplings, where nuclear motion modulates intersite electronic parameters such as hopping and/or spin–orbit terms. In molecular systems, these modes are typically associated with low-frequency torsions between molecular fragments,^47,55^ or with modes that modulate the distance between molecular fragments (e.g., interbase stacking stretchings in DNA). In molecular semiconductors, Peierls modes are central to the nontrivial charge transport rationalized in terms of transient localization.^56^

From the viewpoint of the WL criterion, Peierls coupling is qualitatively different from Holstein coupling, because it can generate path-dependent SU(2) phases and thereby break the site-local spin gauge symmetry discussed in the single-channel case. As a consequence, spin polarization becomes possible already in single-channel models, as first demonstrated numerically in ref 47.

### (1) Vibronic Wilson Loop Picture

We start by considering the tight-binding Hamiltonian for a dimer, and we then generalize to an arbitrary number of sites and modes below. The dimer Hamiltonian reads

14 $$H_{P,2}=\overset{2}{\underset{j=1}{\sum }}\underset{\sigma }{\sum }\epsilon_{j}c_{j\sigma }^{†}c_{j\sigma }+\hbar \omega_{0}\left(a^{†}a+\frac{1}{2}\right)+\underset{\sigma }{\sum }c_{1\sigma }^{†}c_{2\sigma }\left[-t-\frac{g}{\sqrt{2}}\left(a^{†}+a\right)\right]+\mathrm{h.c.}+i\underset{\sigma }{\sum }c_{1\sigma }^{†}\mathrm{v⃗}\cdot \mathrm{\sigma ⃗}c_{2\sigma }\left[\lambda +\frac{\chi }{\sqrt{2}}\left(a^{†}+a\right)\right]+\mathrm{h.c.}$$

where *t* and λ are the purely electronic hopping and SOC amplitudes, while *g* and χ describe the corresponding linear Peierls modulation by a vibrational mode of frequency ω_0_.

The WL viewpoint developed for purely electronic models extends naturally to the *combined electronic–vibrational space*, where Peierls-type couplings generate elementary closed plaquettes. In this setting, the relevant loops are not spatial rings but rather minimal *vibronic* loops formed by sequences of phonon-assisted transitions. In the lowest vibronic sector spanned by *n*_*b*_ = 0, 1, Peierls coupling induces a minimal closed plaquette in vibronic space connecting the four vibronic states (Figure 3):

15 |1,0⟩→|2,1⟩→|1,1⟩→|2,0⟩→|1,0⟩

where the first label denotes the electronic site and the second the vibrational quantum number. The SU(2) phase accumulated around this elementary cycle defines a *vibronic* WL. To make this explicit, we introduce the vibronic bond operator

16 $$T\left(X\right)=-\left[t+gX\right]1+i\left[\lambda +\chi X\right]\mathrm{v⃗}\cdot \mathrm{\sigma ⃗},,X=\frac{a^{†}+a}{\sqrt{2}}$$

which includes a purely electronic contribution

17 $$T\left(X=0\right)\equiv T_{0}=-t1+i\lambda \mathrm{v⃗}\cdot \mathrm{\sigma ⃗}$$

as well as a part linear in *X* ([Disp-formula eq16]),

18 G≡−g1+iχv⃗·σ⃗

**Figure 3:**
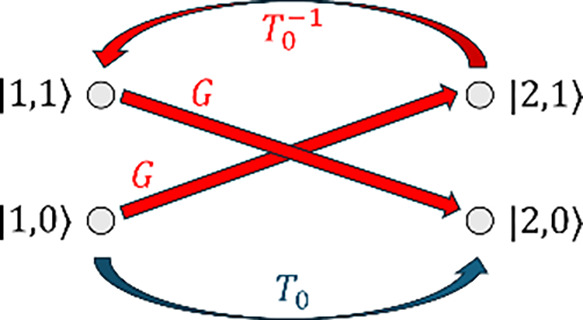
Vibronic plaquette and two-path interferometer induced by Peierls coupling in a dimer. The minimal closed loop in the electronic–vibrational Hilbert space involves the four vibronic states |1, 0⟩, |2, 0⟩, |1, 1⟩, and |2, 1⟩. Straight red arrows denote phonon-assisted vertices *G*, while curved links represent electronic propagation via ${T_{0}}^{-1}$ . The blue curved link corresponds to the direct electronic hop with amplitude *T*_0_, so that the figure can be interpreted as a vibronic two-path interferometer: a direct electronic path and a phonon-assisted trajectory involving two *G* vertices and one ${T_{0}}^{-1}$ . The ordered product of amplitudes around the loop defines the vibronic Wilson loop $W_{vib}=U_{dir}^{†}U_{bos}$ , whose trace controls the interference contribution. Kets label vibronic states, with indices referring to the site and the vibrational quantum number.

In the *n*_*b*_ = 0, 1 truncated space the phonon-assisted bond operator *T*(*X*) is generated by *G*. The elementary vibronic WL associated with the plaquette ([Disp-formula eq15]) is defined as the net SU(2) phase accumulated along the closed vibronic loop generated by the sequence of operators $GT_{0}^{-1}GT_{0}^{-1}$ . Extracting the phase we can write

19 $$W_{vib}\equiv e^{+i\Phi_{vib}\mathrm{n̂}\cdot \mathrm{\sigma ⃗}}$$

where Φ_vib_ represents the total spin rotation acquired around the elementary vibronic plaquette. The explicit evaluation of Φ_vib_ in terms of the microscopic parameters *t*, λ, *g*, and χ is reported in the Supporting Information. A nontrivial $W_{vib}$ signals that no global site-local spin gauge can be defined in the vibronic space, so that spin-dependent interference and hence spin polarization are symmetry-allowed already in a single channel. The explicit evaluation of ([Disp-formula eq19]) shows that the vibronic WL is trivial when

20 $$\Phi_{vib}=2\pi m\;\left(m\in N\right)\Leftrightarrow \frac{g}{t}=\frac{\chi }{\lambda }$$

In this condition, all bond operators are proportional to the same SU(2) matrix,

21 $$T\left(X\right)=f\left(X\right)T_{0},,f\left(X\right)\in R$$

so that spin and vibrational degrees of freedom factorize identically. This factorization holds on the full vibronic Hilbert space: any multiphonon process involving an arbitrary sequence of bond operators satisfies

22 $$T\left(X_{1}\right)\;T\left(X_{2}\right)\;\cdot \cdot \cdot \;T\left(X_{N_{b}}\right)=\left[f\left(X_{1}\right)\;f\left(X_{2}\right)\;\cdot \cdot \cdot \;f\left(X_{N_{b}}\right)\right]T_{0}$$

implying that *all* vibronic trajectories share the same SU(2) rotation. Consequently, the vibronic WL is trivial for any number of phonons *N*_*b*_ and spin polarization is forbidden. Whenever condition ([Disp-formula eq20]) is not satisfied, Peierls coupling generates a genuinely nontrivial vibronic WL, enabling spin polarization.

The same result admits a physical interpretation in terms of a vibronic two-path interferometer. Focusing on transport between the vibronic states |1, 0⟩ and |2, 0⟩, the electron can propagate coherently along two inequivalent trajectories in the combined electronic–vibrational space (Figure 3).

The first contribution corresponds to a direct electronic hopping with amplitude

23 $$A_{dir}\equiv T_{0}=-R_{dir}e^{-i\phi_{dir}\mathrm{n̂}\cdot \mathrm{\sigma ⃗}},,\tan\left(\phi_{dir}\right)=\frac{\lambda ∥\mathrm{v⃗}∥}{t}$$

The second contribution is a phonon-assisted trajectory |1, 0⟩ → |2, 1⟩ → |1, 1⟩ → |2, 0⟩, with amplitude

24 $$A_{bos}\equiv GT_{0}^{-1}G=R_{dir}R_{bos}e^{+i\phi_{bos}\mathrm{n̂}\cdot \mathrm{\sigma ⃗}}$$

where *R*_bos_ and ϕ_bos_ follow from the closed-form evaluation reported in the Supporting Information. The relative SU(2) phase accumulated by the two trajectories is precisely the vibronic WL defined in eq 19, Accordingly, the spin-averaged interference term in the transition probability reads

25 $$I_{int}\propto \cos\left(\Phi_{vib}\right)=\cos\left(\phi_{bos}+\phi_{dir}\right)$$

showing explicitly that a nontrivial vibronic WL produces spin-dependent interference between the two paths. When $W_{vib}=1$ , the two amplitudes differ only by a global SU(2) phase and transport is spin independent.

### (2) Two-Site Model: Gauge Analysis

We now clarify how the nontriviality of the vibronic Wilson loop $W_{vib}$ is directly connected to the breakdown of a site-local spin gauge, and how the symmetry-restoring condition *g*/*t* = χ/λ emerges. This connection can be made fully explicit in the minimal two-site model.

In the presence of Peierls coupling, the electron–phonon interaction modulates both the spin-independent hopping and the spin–orbit terms. As a consequence, the bond operator cannot in general be factorized into a scalar prefactor times a fixed SU(2) matrix, in contrast to the Holstein case discussed above. A detailed diagonalization and Lang–Firsov analysis (reported in the Supporting Information) shows that, after integrating out the phonons, the effective two-site Hamiltonian retains a spin-dependent hopping term that cannot be removed by a site-local SU(2) rotation.

In particular, the effective spin–orbit coupling takes the form

26 $$\mathrm{\lambda ̃}\overset{\sim }{\mathrm{v⃗}}\propto \left(\lambda g-\chi t\right)\mathrm{v⃗}$$

so that all components of $\mathrm{\lambda ̃}\overset{\sim }{\mathrm{v⃗}}$ vanish simultaneously if and only if

27 $$\frac{g}{t}=\frac{\chi }{\lambda }$$

On this symmetry-restoring manifold the bond operator factorizes, a site-local spin gauge can be defined, and spin polarization is forbidden. Away from this condition, the gauge is broken and spin polarization becomes symmetry-allowed already in the minimal single-channel setting.

### (3) Extension to an Arbitrary Number of Sites and Modes

The vibronic Wilson-loop criterion is not restricted to the two-site model but extends naturally to arbitrary chain lengths. In realistic molecular systems, several Peierls-active vibrational modes may couple to the same bond. For an *N*-site single-channel tight-binding chain with multiple Peierls modes, denoted by the index μ, Peierls coupling generically generates nontrivial vibronic Wilson loops unless the phonon-induced modulations of the spin-independent and spin-dependent hopping amplitudes are proportional on every bond and for every vibrational mode,

28 $$\frac{g_{j,\mu }}{t_{j}}=\frac{\chi_{j,\mu }}{\lambda_{j}},\forall j,\mu$$

Only on this symmetry-restoring manifold do all vibronic trajectories acquire the same SU(2) rotation, preserving a site-local spin gauge, and forbidding spin polarization. Any deviation from this condition breaks the gauge and enables the CISS. The full derivation for arbitrary chain lengths and multiple Peierls modes is provided in the Supporting Information.

### Numerical Benchmark

The emergence of a nonzero spin polarization in the unitary time evolution constitutes a necessary condition for observing finite spin selectivity in any experimental transport or photoemission setup. For this reason, we benchmark the WL and gauge-symmetry criteria by performing explicit numerical simulations first of the unitary dynamics induced by the different model Hamiltonians analyzed in this work.

Since a realistic modeling of CISS requires open quantum system approaches, we then perform further numerical simulations considering the simplest open quantum system in which CISS was observed.^11–13^ This corresponds to an electron transfer setup, where the chiral molecule (described by the Hamiltonians analyzed above) is connected incoherently to electron donor (D) and electron acceptor (A) sites. This situation recalls a two-terminal electron-transport setup and hence allow us to draw rather general conclusions.

### (1) Unitary Dynamics

We start by simulating the real-time unitary evolution of a single electron coupled to vibrational modes and monitor the local observables ⟨*S*_*z*,*i*_(*t*)⟩. Representative results are shown in the main text, while full numerical details—including basis truncation, and time-propagation schemes—are reported in Supporting Information.

The simulations shown in Figure 4 confirm all analytical predictions:

(i) *Single-channel models*. Single-channel chains without vibrations, as well as chains coupled to Holstein modes, show vanishing spin polarization at all times within numerical precision (Figure 4-1a and Figure 4-2a). This remains true even in the presence of several independent Holstein modes, in agreement with the analytical result that Holstein coupling yields trivial WLs and preserves site-local spin gauge symmetry.
(ii) *Peierls coupling.* Introducing Peierls-type electron–vibration coupling immediately leads to a finite spin polarization in the unitary dynamics (Figure 4-3a). This holds both for single-Peierls-mode models and multi-Peierls-mode models, confirming that bond-modulating vibrations generically break the site-local spin gauge. The polarization vanishes again when *g*_*j*,μ_/*t*_*j*_ = χ_*j*,μ_/λ_*j*_ is imposed, in agreement with the analytical criterion (Figure 4–4a).
(iii) *Multiple transport channels*. Models with multiple transport channels (e.g., NN hopping combined with NNN SOC) display finite spin polarization even in the absence of vibrations, as expected from the WL analysis carried out. Adding Peierls coupling generally enhances the magnitude of the polarization (Figure 4-3a and Figure 4-3b).

To visualize how Peierls couplings control the polarization signal, we scanned the spin-independent and spin-dependent couplings (*g*, χ) and computed, for each site *k* = 1, ..., 6, the peak polarization

29 $$P_{z,k}^{\max}\left(g,\chi \right)=2\underset{t\in \left[0,1\right]ps}{\max}\left|\left\langle S_{z,k}\left(t\right)\right\rangle \right|$$

as shown in Figure 5. We used degenerate on-site energies, NN hopping *t* = 0.1 eV, NNN SOC λ = 10^–3^ eV with v⃗∥ẑ , and truncated the bosonic basis to *N*_*b*_ = 50.

**Figure 4:**
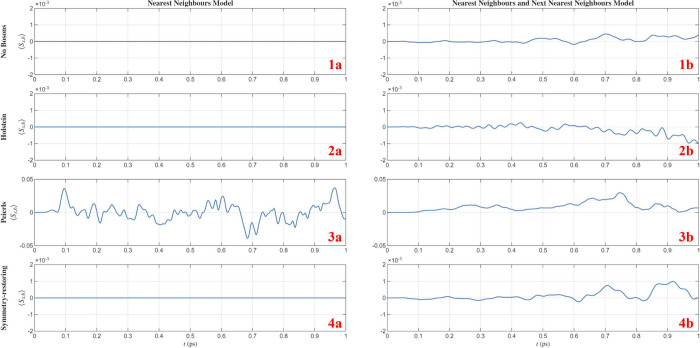
Time evolution of the spin polarization along the *x* axis at the site *N*, ⟨*S*_*x*,*N*_(*t*)⟩, for different coupling schemes. (**1**) The left column corresponds to the single-channel model (nearest-neighbor, NN). (**2**) The right column shows the multichannel case including next-nearest-neighbor (NN+NNN) interactions. From top to bottom: (**1**) no bosonic coupling, (**2**) Holstein-type electron–boson coupling, (**3**) Peierls-type coupling, and (**4**) the symmetry-restoring configuration. The vertical scale is the same for the first, second, and fourth rows, whereas the third row (Peierls case) uses a larger range ([−0.05, 0.05]) for visual clarity.

**Figure 5:**
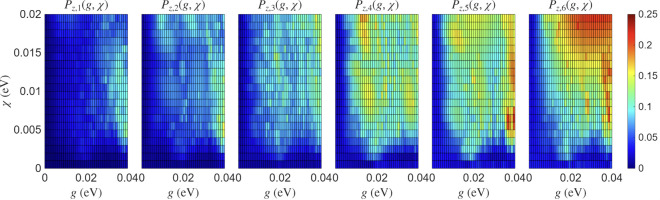
Peak spin polarization maps $P_{z,k}^{\max}\left(g,\chi \right)$ for a chain with *N* = 6 sites and Peierls coupling to a single mode with *ℏω*_0_ = 50 meV. We use degenerate on-site energies, NN hopping *t* = 0.1 eV, NNN SOC λ = 10^–3^ eV with v⃗=(0,0,1) , and no Holstein coupling; the boson truncation is *N*_*b*_ = 50. Axes: *g* ∈ [0, 0.04] eV (horizontal) and χ ∈ [0, 0.02] eV (vertical). Color encodes $P_{z,k}^{\max}\left(g,\chi \right)=2\max_{t\in \left[0,1\right]ps}\left|\left\langle S_{z,k}\left(t\right)\right\rangle \right|$ .

The polarization systematically increases with the site index, reaching values $P_{z,k}^{\max}$ ∼ 0.2–0.25 at the end of the chain, and grows monotonically with increasing χ at fixed *g*. Changing the vibrational energy from *ℏω*_0_ = 50 to 100 meV leaves the qualitative structure of the maps unchanged (see the Supporting Information), indicating robustness with respect to the phonon frequency.

Overall, the numerical simulations fully support the WL analysis: spin polarization arises if and only if the site-local spin gauge is broken, either by multiple transport channels or by Peierls-type electron–vibration coupling. When the gauge is preserved, the unitary dynamics remains strictly spin independent.

### (2) Extension to Open-System Dynamics

To assess the robustness of these conclusions beyond the unitary regime, we now extend the numerical analysis to an open-system setting, including incoherent injection from D to the chiral bridge and extraction from the bridge to A, besides decoherence processes on the bridge.^57^ Charge transfer between donor, bridge, and acceptor is described with the Redfield master equation, following standard approaches to electron-transfer dynamics. The reduced density matrix ρ evolves as

30 $$\hbar \frac{d\rho }{dt}=-i\left[H_{\mathrm{tot}},\rho \right]+\Gamma \underset{\xi =D,A}{\sum }\left(Y_{\xi }\rho X_{\xi }^{†}-X_{\xi }^{†}Y_{\xi }\rho +\mathrm{h.c.}\right)+\Gamma_{d}\underset{n}{\sum }\underset{\mu =x,y,z}{\sum }\left(L_{n\mu }\rho L_{n\mu }^{†}-\frac{1}{2}\left\{L_{n\mu }^{†}L_{n\mu },\rho \right\}\right)$$

where the first term describes the coherent evolution, the second term accounts for incoherent transfer processes between donor, bridge, and acceptor, and the third term accounts for generic spin decoherence, as assumed in.^58^

The operators *X*_*D*_ and *X*_*A*_ describe spin-independent electron transfer $X_{D}=\underset{\sigma }{\sum }c_{B,\sigma }^{†}c_{D,\sigma }$ and $X_{A}=\underset{\sigma }{\sum }c_{A,\sigma }^{†}c_{B,\sigma }$ , while *Y*_ξ_ encode the spectral properties of the environment and, in the wide-band and low-temperature limit considered here, reduce to projectors onto energetically allowed transitions; see ref 38. Finally, the operators $L_{n\mu }=c_{B,n,\sigma }^{†}\sigma_{\mu }c_{B,n,\sigma }$ describe local spin decoherence channels on the bridge sites, with Γ_*d*_ controlling the decoherence strength. We initialize the system with an electron on the donor as a mixture of spin-up and spin-down and monitor the spin polarization at the acceptor site,

31 $$P_{A}\left(t\right)=\left\langle n_{A,↑}-n_{A,↓}\right\rangle$$

as a function of time.

Simulations are shown in Figure 6 for the 4-site system in the different regimes examined in this work, without and with decoherence (left and right columns). These results show that the qualitative behavior identified by the WL loop analysis and by the unitary dynamics is preserved: systems with trivial Wilson loop do not develop any spin polarization, while nontrivial Wilson loops lead to finite spin polarization also in the presence of decoherence. In general, decoherence reduces the oscillation amplitude of the spin polarization on the bridge and stabilizes it at the acceptor site.

**Figure 6:**
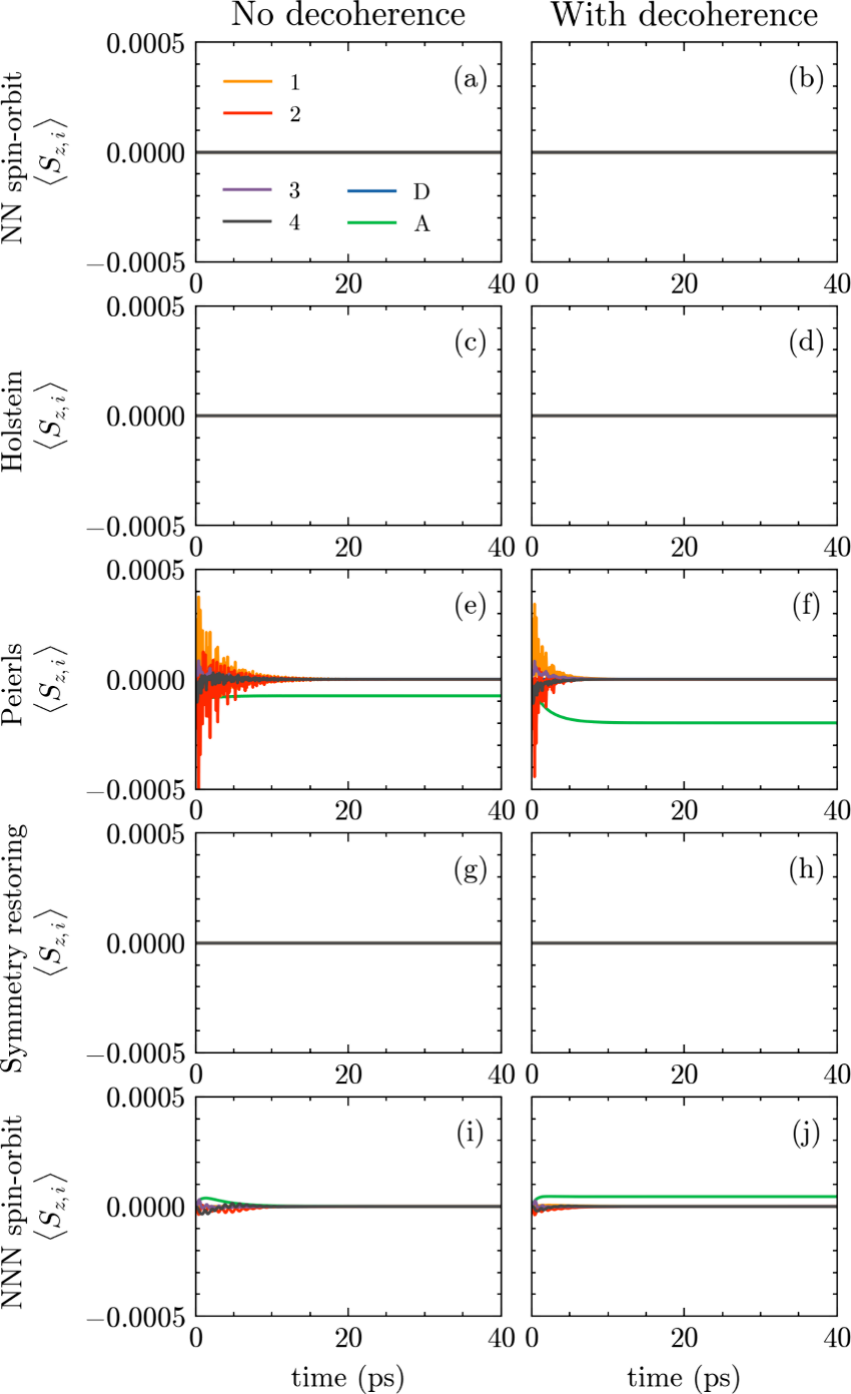
Time evolution of the spin polarization along the *z* axis at the site *N*, ⟨*S*_*z*,*N*_(*t*)⟩, for different coupling schemes, as described in the main text. (a,b) purely electronic model with only NN interactions. (c,d) The same as in (a,b), but adding a Holstein mode. (e,f) The same as in (a,b), but adding a Peierls mode. (g,h) The same as in (e,f), but in the symmetry restoring condition, setting $g_{i}^{\mathrm{Peierls}}=1/10t_{i}$ and χ_*i*_ = 1/10λ_*i*_. (i,j) a purely electronic model with NNN SOC. The transfer rate is set to Γ = 5 × 10^–5^ eV. The decoherence rate is set to Γ_*d*_ = 0 (left column) and Γ_*d*_ = 1 × 10^–6^ eV (right column). Four bridge sites are considered in these calculations, using the parameters reported in the Supporting Information. Specifically, the first 4 values for ϵ and *g* ^Holstein^, the first three for *t*, λ, *g* ^Peierls^, χ, *v*_*x*_, *v*_*y*_ and *v*_*z*_. In the case of next-nearest neighbor SOC, the first two values are instead used for λ, χ, *v*_*x*_, *v*_*y*_ and *v*_*z*_.

Hence, the Wilson loop condition remains a robust indicator for the emergence of spin polarization also in an open quantum system framework.

### Discussion and Outlook

The central message of this work is the definition of a necessary condition for the emergence of CISS in electronic and vibronic tight binding models: spin polarization can arise only when a nontrivial WL appears. In this case, the accumulated spin rotation along distinct electronic or vibronic paths cannot be removed, spin-dependent interference becomes intrinsic and manifests as CISS. When all WLs are instead trivial, then spin polarization necessarily vanishes. This WL formulation provides a unifying framework that brings together several seemingly disparate mechanisms proposed in the literature and reduces them to a common topological criterion.

Beyond its conceptual implications, this Letter suggests practical design principles for molecular and nanoscale systems. Spin selectivity is favored in structures where vibrations modulate intersite couplings, where electrons can propagate along multiple inequivalent paths (due to different hopping or SOC connectivities) or where several orbital channels participate in transport. Conversely, situations in which all spin-dependent processes act on the same bonds are expected to suppress the CISS. Our numerical simulations confirm these predictions, both in the unitary regime and in the presence of incoherent processes and dephasing.

This work naturally leads to several additional developments. A first extension concerns electron–electron interactions. While our analysis focuses on single-electron dynamics, interactions are known to play an important role in molecular systems and have been proposed as an amplification mechanism for CISS. It will be interesting to investigate how the WL criterion generalizes in the presence of many-body correlations and whether interaction-induced effective gauge fields can generate or enhance nontrivial spin-dependent loops.

A second open question is whether conditions for perfect *spin filtering* can be achieved within vibronic models. In purely electronic ring geometries, it has been shown that suitably tuned external fields can lead to complete suppression of one spin component, yielding ideal spin filters.^59^ An intriguing possibility raised by our results is that Peierls-type vibrations might play an analogous role, enabling vibration-controlled perfect filtering through nontrivial vibronic WLs even in the absence of external magnetic fields.

Finally, dissipation and decoherence represent crucial ingredients for connecting transient unitary dynamics to experimentally observed steady-state signals. Within the present framework, we find that incoherent processes do not modify the symmetry-based WL criterion: they do not generate spin selectivity in systems with trivial WL, nor destroy it in systems with nontrivial WL. More realistic descriptions including electrodes, interfacial effects, or many-body correlations may further enhance or modify the observed spin polarization, but the WL provides a minimal and robust criterion for identifying when spin-dependent interference is symmetry allowed. Addressing these aspects will be essential to bridging the gap between minimal theoretical models and realistic descriptions of CISS experiments.

## Supplementary Material



## Notes

Single transport channel fermionic chains with equal range hopping and SOC terms, Wilson-loop interpretation in the multiple transport channels case, single transport channel chains and Holstein vibrations, Peierls coupling and the breaking of the spin gauge, Wilson-loop interpretation with Peierls bosons, extension of the vibronic Wilson-loop analysis to arbitrary chains and multiple modes, derivation of the Wilson loop in coherent dynamics, numerical verification for coherent evolution; figures of time evolution of the spin polarization, peak spin polarization maps, (PDF)
