# Supplementary material for: Wilson Loop as a Tool to Investigate Chirality-Induced Spin Selectivity: Role of Vibrations and Multiple Channels
Source: J Phys Chem Lett. 2026 May 9;17(20):5629–38. doi: 10.1021/acs.jpclett.6c00585 (PMC13200249; doi:10.1021/acs.jpclett.6c00585)
Supplement: Supplementary file 1 [file jz6c00585_si_001.pdf]

Supporting Information to
Wilson Loop as a tool to investigate Chirality-Induced Spin Selectivity:
role of vibrations and multiple channels

L. Celada,[1,2] D.K.A. Phan Huu,[1] A. Chiesa,[1,2,3] P. Santini,[1,2,3] L. Griguolo,[1,4] and S. Carretta[1,2,3,*]

[1]*Dipartimento di Scienze Matematiche, Fisiche e Informatiche, Università di Parma, Parco Area delle Scienze, 53/A, I-43124 Parma, Italy.*

[2]*INFN–Sezione di Milano-Bicocca, gruppo collegato di Parma, 43124 Parma, Italy*

[3]*Consorzio Interuniversitario Nazionale per la Scienza e Tecnologia dei Materiali (INSTM), I-50121 Firenze, Italy*

[4]*Gruppo Collegato di Parma, INFN-Sezione Milano-Bicocca, I-43124 Parma, Italy.*

* stefano.carretta@unipr.it

I. SINGLE TRANSPORT CHANNEL FERMIONIC CHAINS WITH EQUAL-RANGE HOPPING AND SOC TERMS

We start with

$$H_0 = \sum_{j=1}^{N}\sum_{\sigma} \epsilon_j c^\dagger_{j\sigma} c_{j\sigma} - \sum_{j=1}^{N-1}\sum_{\sigma} \Big[t_j\, c^\dagger_{j\sigma} c_{j+1\sigma} - i\lambda_j\, c^\dagger_{j\sigma} \vec{v}_j \cdot \vec{\sigma} c_{j+1\sigma} + \text{h.c.}\Big]. \tag{S1}$$

where:

$$\vec{v}_j \cdot \vec{\sigma} = v_{x,j}\sigma_x + v_{y,j}\sigma_y + v_{z,j}\sigma_z \tag{S2}$$

Comments on vector $\vec{v}$

A possible choice for the vector $\vec{v}_j$ is the one associated with a helical molecular structure having a single turn, with radius a, pitch c, and site positions

$$\vec{r}_j = [a\cos((j-1)2\pi/N),\, a\sin((j-1)2\pi/N),\, (j-1)c/(N-1)]. \tag{S3}$$

Within this description, the local vector $\vec{v}_j$ can be defined as $\vec{v}_j = \vec{d}_{j+1} \times \vec{d}_{j+2}$, where $\vec{d}_{j+s} = (\vec{r}_j - \vec{r}_{j+s})/|\vec{r}_j - \vec{r}_{j+s}|$, as in Ref. [1]. With these definitions, changing the molecular enantiomer corresponds to the transformation $(v_{x,j}, v_{y,j}, v_{z,j}) \to (-v_{x,j}, v_{y,j}, -v_{z,j})$.

More generally, $\vec{v}_j$ can be regarded as a phenomenological vector that weights differently the components of the spin–orbit coupling (SOC), thus allowing for anisotropic or site-dependent SOC effects within the same formalism.

Finally, the specific form of $\vec{v}_j$ is not relevant for the analytical calculations that follow, and will only be used in the numerical simulations.

Consider the combined hopping matrix between site j and $j+1$ in spin space,

$$T_j \equiv -t_j \mathbf{1}_{2\times2} + i\lambda_j \vec{v}_j \cdot \vec{\sigma} = -t_j \mathbf{1}_{2\times2} + i\Lambda_j \hat{n}_j \cdot \vec{\sigma}, \tag{S4}$$

where we have defined

$$\Lambda_j \equiv \lambda_j ||\vec{v}_j||, \qquad \hat{n}_j \equiv \frac{\vec{v}_j}{||\vec{v}_j||}. \tag{S5}$$

Now it is true

$$e^{-i\phi_j \hat{n}_j\cdot\vec{\sigma}} = \cos(\phi_j)\; \mathbf{1}_{2\times2} - i\sin(\phi)\hat{n}_j \cdot \vec{\sigma}, \tag{S6}$$

So we define

$$R_j \equiv \sqrt{t_j^2 + \Lambda_j^2}, \qquad \phi_j \equiv \arctan(\Lambda_j/t_j). \tag{S7}$$

it follows immediately that

$$T_j = -R_j\, U_j, \tag{S8}$$

with U_j the unitary, site-dependent spin rotation

$$U_j = e^{-i\phi_j \hat{n}_j\cdot\vec{\sigma}} \tag{S9}$$

Now we define recursively

$$W_1 = \mathbf{1}_{2\times 2} \qquad W_{j+1} = U_j^{-1} W_j = U_j^\dagger W_j = U_j^\dagger \left(\prod_{\ell=1}^{j-1} U_\ell\right)^{-1} \tag{S10}$$

which is unitary as well. Define rotated fermions $d_{j,\sigma} = W_j^\dagger c_{j,\sigma}$. Under this transformation the inter-site hopping becomes

$$\begin{aligned}
c_{j,\sigma}^\dagger T_j c_{j+1,\sigma} &= c_{j,\sigma}^\dagger \left(-R_j\, U_j\right) c_{j+1,\sigma} \\
&= c_{j,\sigma}^\dagger W_j W_j^\dagger \left(-R_j\, U_j\right) W_{j+1} W_{j+1}^\dagger c_{j+1,\sigma} \\
&= d_{j,\sigma}^\dagger W_j^\dagger \left(-R_j\, U_j\right) W_{j+1} d_{j+1,\sigma} \\
&= -R_j d_{j,\sigma}^\dagger W_j^\dagger U_j W_{j+1} d_{j+1,\sigma} \\
&= -R_j d_{j,\sigma}^\dagger W_j^\dagger U_j U_j^{-1} W_j d_{j+1,\sigma} \\
&= -R_j d_{j,\sigma}^\dagger W_j^\dagger W_j d_{j+1,\sigma} \\
&= -R_j d_{j,\sigma}^\dagger d_{j+1,\sigma}
\end{aligned} \tag{S11}$$

so the transformed Hamiltonian is spin independent,

$$\tilde{H} = \sum_{j=1}^{N} \sum_\sigma \epsilon_j d_{j,\sigma}^\dagger d_{j,\sigma} - \sum_{j=1}^{N-1} R_j \sum_\sigma \left(d_{j,\sigma}^\dagger d_{j+1,\sigma} + \text{h.c.}\right). \tag{S12}$$

As we can see the Hamiltonian is no longer spin-dependent and therefore we cannot have any polarization.

A. Ring toy-model: interpretation as an interferometer

The role of the Wilson loop becomes particularly transparent in presence of periodic boundary conditions, where electron propagation naturally involves closed paths. In this case, the

Wilson loop directly captures the total SU(2) spin rotation accumulated by an electron after a complete passage around the system, and provides a physical interpretation in terms of quantum interference.

For periodic boundary conditions the site-dependent SU(2) rotation that in the open chain removes the spin dependence generally fails to eliminate it on the closing bond. After the transformation, the hopping term that connects site N to site 1 becomes

$$\begin{aligned} c^\dagger_{N,\sigma} T_N c_{N+1,\sigma} &= c^\dagger_{N,\sigma} T_N c_{1,\sigma} \\ &= d^\dagger_{N,\sigma} W_N^\dagger \left(-R_N\, U_N\right) W_1 d_{1,\sigma} \\ &= -R_N d^\dagger_{N,\sigma} W_N^\dagger U_N W_1 d_{1,\sigma} \end{aligned} \tag{S13}$$

we evaluate

$$W_N^\dagger U_N W_1 = \left(\prod_{\ell=1}^{N-1} U_\ell\right) U_N = \prod_{\ell=1}^{N} U_\ell = \mathcal{W} \in \mathrm{SU}(2) \tag{S14}$$

which enters (S13) as

$$-R_N\, d^\dagger_{N,\sigma}\, \mathcal{W}\, d_{1,\sigma}. \tag{S15}$$

The matrix $\mathcal{W}$ is the Wilson loop: it captures the total spin rotation accumulated by an electron after completing one loop. If $\mathcal{W} = \mathbf{1}$ the transformed Hamiltonian is fully spin independent, while for $\mathcal{W} \neq \mathbf{1}$ a global SU(2) twist remains and the system can retain spin polarization. We can make $\mathcal{W} = 1$ only for a specific choice of parameters. Let us set

$$\begin{aligned} &\lambda_j = \lambda\,, \quad t_j = t\,, \quad \vec{v}_j = \vec{v}\,, \\ &\Lambda_j = \Lambda\,, \quad \phi_j = \phi\,, \quad R_j = R \quad \forall j. \end{aligned} \tag{S16}$$

Then

$$\mathcal{W} = \prod_{j=1}^{N} U_j = U^N = e^{-iN\phi \hat{n}\cdot\vec{\sigma}}\,, \tag{S17}$$

in this case, the Wilson Loop is the identity if

$$\begin{aligned} &N\phi = 2\pi m \quad \text{with} \quad m \in \mathbb{N}\,, \\ &\lambda ||\vec{v}|| = t \cdot \tan\left(\frac{2\pi m}{N}\right). \end{aligned} \tag{S18}$$

This result depends only on the total SU(2) rotation around the ring and is independent of the particular path used to compute the Wilson loop.

The appearance of the Wilson loop can be understood from a simple interference picture. In a ring geometry, an electron injected at a given site can reach another site through two inequivalent lattice routes, clockwise and counter-clockwise, exactly as a single particle in a double-slit experiment reaches the detector through distinct paths. Because the propagation of a quantum particle is determined by the coherent superposition of all allowed paths, the electron effectively interferes with itself: the total amplitude is the sum of the contributions associated with the two trajectories. Any phase accumulated along either route therefore has observable consequences, and the relative phase between the clockwise and counter-clockwise paths determines the interference pattern. A similar path-interference approach has been adopted in the context of spin–orbit coupled tight-binding circuits and rings, where the spin dynamics is encoded in ordered products of SU(2) hopping matrices associated with different lattice paths. In particular, Hatano *et al.* [2] showed that spin-dependent interference effects can be fully captured by combining the SU(2) transport operators accumulated along inequivalent paths and that the resulting interference is governed by the net SU(2) phase acquired around a closed loop. The physical meaning of the Wilson loop becomes transparent by considering the closed chain as a two-path interferometer. In the ring geometry, an electron initially at site i can reach site j (with $1 < i < j < N$ and $j - i = \ell$) through two inequivalent trajectories, as illustrated in Fig. S1: a clockwise path described by $U_+ = (-RU)^\ell$ (blue arrows) and a counter-clockwise path described by $U_- = \left((-RU)^{N-\ell}\right)^{-1}$ (red arrows). The operators associated with clockwise and counter-clockwise propagation between two sites separated by ℓ bonds are, respectively,

$$U_+ = \prod_{k=i}^{\ell-1} -R_k U_k = (-RU)^\ell, \qquad U_- = \left((-RU)^{N-\ell}\right)^{-1} = (-RU)^{\ell-N}. \tag{S19}$$

The total propagation amplitude of a spinor transported from site i to j is therefore

$$\mathcal{A}_{\rm tot}|\psi\rangle = (U_+ + U_-)\,|\psi\rangle = (RU)^\ell \left[\mathbf{1} + (-R)^{-N}\mathcal{W}^{-1}\right]|\psi\rangle. \tag{S20}$$

The matrix $\mathcal{W}$ that appears here is precisely the Wilson loop defined in Eq. (S17), and plays the role of the SU(2) phase difference between the two paths. To quantify the interference between the two paths, we evaluate the squared norm of the total amplitude:

$$\begin{aligned} ||\mathcal{A}_{\rm tot}|\psi\rangle||^2 &= \langle\psi|\,(U_+ + U_-)^\dagger (U_+ + U_-)\,|\psi\rangle \\ &= \langle\psi|\big((-R)^{2\ell}\left[\mathbf{1} + (-R)^{-2N}\mathbf{1} + (-R)^{-N}[\mathcal{W}^{-1} + \mathcal{W}]\right]\big)|\psi\rangle, \end{aligned} \tag{S21}$$

The last term encode the coherent interference between the clockwise and counter-clockwise paths:

$$I_{\text{int}}(\psi) = \langle\psi|\big(U_+^\dagger U_- + U_-^\dagger U_+\big)|\psi\rangle = \langle\psi|(-R)^{2\ell-N}[\mathcal{W}^{-1} + \mathcal{W}]|\psi\rangle. \tag{S22}$$

Averaging over spin (i.e., taking the trace over spin space) gives

$$\begin{aligned} I_{\text{int}} &= \langle I_{\text{int}}(\psi)\rangle_{\text{spin}} \\ &= \frac{(-R)^{2\ell-N}}{2}\text{Tr}\big[\mathcal{W}^{-1} + \mathcal{W}\big]\,. \end{aligned} \tag{S23}$$

We now insert the explicit form of the Wilson loop:

$$\mathcal{W} = e^{-iN\phi\,\hat{n}\cdot\vec{\sigma}} = \cos(N\phi)\,\mathbf{1} - i\sin(N\phi)\,(\hat{n}\cdot\vec{\sigma}), \tag{S24}$$

$$\mathcal{W}^{-1} = e^{+iN\phi\,\hat{n}\cdot\vec{\sigma}} = \cos(N\phi)\,\mathbf{1} + i\sin(N\phi)\,(\hat{n}\cdot\vec{\sigma}). \tag{S25}$$

Since $\text{Tr}[\mathbf{1}] = 2$ and $\text{Tr}[\hat{n}\cdot\vec{\sigma}] = 0$, one finds

$$\text{Tr}\big[\mathcal{W}^{-1} + \mathcal{W}\big] = 4\cos(N\phi). \tag{S26}$$

Hence, the spin-averaged interference term becomes

$$I_{\text{int}} = 2(-R)^{2\ell-N}\cos(N\phi)\,, \tag{S27}$$

and the

$$\big\langle\|\mathcal{A}_{\text{tot}}\|^2\big\rangle_{\text{spin}} = (-R)^{2\ell} + (-R)^{2\ell-N} + 2(-R)^{2\ell-N}\cos(N\phi)\,. \tag{S28}$$

This result shows that the interference between the two propagation paths is entirely governed by the SU(2) phase accumulated after one full loop around the ring, encoded in the Wilson loop $\mathcal{W}$. Constructive (destructive) interference occurs for $N\phi = 2\pi m$ ($N\phi = \pi(2m{+}1)$), corresponding to $\mathcal{W} = \pm\mathbf{1}$, for which the transport is spin-independent. For intermediate values of ϕ, $\mathcal{W}$ represents a non-trivial SU(2) rotation, leading to spin-dependent interference.

In this sense, the Wilson loop plays the role of a spin analogue of an Aharonov–Bohm phase, encoding the relative spin rotation accumulated along the two directions of motion. Therefore, the condition under which the ring Hamiltonian becomes spin-independent ($N\phi = 2\pi m$) is not accidental: it simply means that the total spin rotation picked up along one full loop is zero, which corresponds to completely constructive interference between the two paths. From this perspective, a single-channel ring with periodic boundary conditions

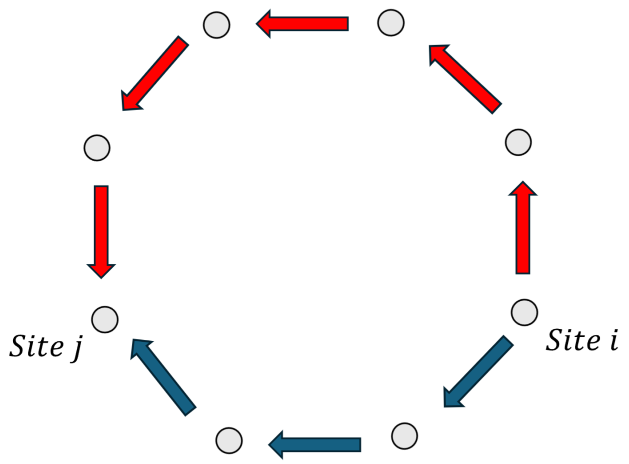

Figure S1: Schematic representation of the two inequivalent propagation paths in a ring geometry: clockwise (red arrows) and counter-clockwise (blue arrows). Their SU(2) phase difference is quantified by the Wilson loop $\mathcal{W}$.

effectively behaves as a two-path interferometer. Even though the underlying lattice is one-dimensional, periodic boundary conditions give rise to two inequivalent propagation paths (clockwise and counter-clockwise), whose SU(2) phase difference is quantified by the Wilson loop. Our SU(2) Wilson loop view directly connects to the literature on equilibrium persistent spin currents in systems with periodic boundary conditions and Rashba SOC terms [3]. In our lattice language this phase is precisely the Wilson loop $\mathcal{W} = U^N$, and the spin-averaged interference term $2\cos(N\phi)$ captures the same physics. Hence, $\mathcal{W} = \pm\mathbf{1}$ corresponds to spin-independent transport (no polarization), while a non-trivial $\mathcal{W}$ produces spin-dependent interference and, in ring geometries, an equilibrium persistent spin current.

II. WILSON-LOOP INTERPRETATION IN THE MULTIPLE TRANSPORT CHANNELS CASE

We explicitly evaluate the interference between the two paths connecting sites j and $j+2$ when nearest-neighbor (NN) hopping and next-nearest-neighbor (NNN) spin–orbit coupling (SOC) coexist. For simplicity we take uniform couplings and a single spin–orbit vector $\vec{v}$:

$$t_j = t, \quad \lambda_j = \lambda, \quad \vec{v}_j = \vec{v}, \quad \hat{n} = \frac{\vec{v}}{\|\vec{v}\|}. \tag{S29}$$

From site j to $j+2$ the electron can propagate via:

(i) two consecutive NN hoppings: $\mathcal{A}_{\rm NN} = (-t\,\mathbf{1})^2 = t^2\,\mathbf{1}$,

(ii) a single direct NNN spin–orbit step: $\mathcal{A}_{\rm NNN} = i\lambda\,(\vec{v}\cdot\vec{\sigma}) = -R\,U$ with $R = \lambda\|\vec{v}\|$ and $U = e^{-i\frac{\pi}{2}\hat{n}\cdot\vec{\sigma}}$.

The total amplitude is their coherent sum,

$$\mathcal{A}_{\rm tot} = t^2\,\mathbf{1} - R\,U. \tag{S30}$$

a. Interference term. The spin-averaged intensity is

$$\frac{1}{2}\mathrm{Tr}\Big[\mathcal{A}_{\rm tot}\mathcal{A}_{\rm tot}^{\dagger}\Big] = \frac{1}{2}\mathrm{Tr}\big[t^4\,\mathbf{1} + R^2\,\mathbf{1} - t^2 R\,(U+U^{\dagger})\big]\,. \tag{S31}$$

Since $U = e^{-i(\pi/2)\hat{n}\cdot\vec{\sigma}} = -i\,\hat{n}\cdot\vec{\sigma}$, one has $U + U^{\dagger} = 0$, and thus the cross (interference) term vanishes exactly:

$$I_{\rm int} = -\frac{t^2 R}{2}\mathrm{Tr}[U + U^{\dagger}] = 0. \tag{S32}$$

The total intensity reduces to

$$\frac{1}{2}\mathrm{Tr}\Big[\mathcal{A}_{\rm tot}\mathcal{A}_{\rm tot}^{\dagger}\Big] = t^4 + R^2 = t^4 + (\lambda\|\vec{v}\|)^2. \tag{S33}$$

b. Local Wilson loop. The absence of interference can be traced back to the non-trivial SU(2) flux enclosed by the elementary triangular loop

$$\mathcal{W}_j^{\triangle} = U_{j,j+1}U_{j+1,j+2}\big(U_{j,j+2}\big)^{-1}. \tag{S34}$$

For this uniform model,

$$U_{j,j+1} = U_{j+1,j+2} = \mathbf{1}, \qquad U_{j,j+2} = U = e^{-i\frac{\pi}{2}\hat{n}\cdot\vec{\sigma}}, \tag{S35}$$

and hence

$$\mathcal{W}_j^{\triangle} = e^{i\frac{\pi}{2}\hat{n}\cdot\vec{\sigma}}, \qquad \mathrm{Tr}\,\mathcal{W}_j^{\triangle} = 2\cos\Big(\frac{\pi}{2}\Big) = 0. \tag{S36}$$

This shows that each local triangle carries a maximally non-trivial SU(2) flux and cannot be made gauge-trivial by any site-local spin rotation. The vanishing of the interference term $I_{\rm int}$ is therefore a direct manifestation of the SU(2) flux piercing the local plaquette.

III. SINGLE TRANSPORT CHANNEL CHAINS AND HOLSTEIN VIBRATIONS

We start with

$$H_{\text{Hol}} = H_0 + \hbar\omega_0\left(a^\dagger a + \frac{1}{2}\right) + \sum_{j=1}^{N} g_j (a^\dagger + a)\sum_{\sigma} c^\dagger_{j\sigma} c_{j\sigma}. \tag{S37}$$

We apply the Schrieffer-Wolff transformation with the following ansatz:

$$\mathbf{S} = \sum_{j=1}^{N}\sum_{\sigma=\uparrow,\downarrow} \frac{g_j}{\hbar\omega_0} c^\dagger_{j,\sigma} c_{j,\sigma}\left(a^\dagger - a\right). \tag{S38}$$

We immediately observe that $\mathbf{S}^\dagger = -\mathbf{S}$ and hence $e^{\mathbf{S}} = \left(e^{-\mathbf{S}}\right)^\dagger$. The unitary transformation is $\tilde{H}_{\text{Hol}} = e^{\mathbf{S}} H_{\text{Hol}} e^{-\mathbf{S}}$.

The transformed operators are:

$$\tilde{a} = a - \sum_{j=1}^{N}\sum_{\sigma=\uparrow,\downarrow} \frac{g_j}{\hbar\omega_0} c^\dagger_{j,\sigma} c_{j,\sigma} \qquad \tilde{a}^\dagger = a^\dagger - \sum_{j=1}^{N}\sum_{\sigma=\uparrow,\downarrow} \frac{g_j}{\hbar\omega_0} c^\dagger_{j,\sigma} c_{j,\sigma} \tag{S39}$$

$$\tilde{c}_{j,\sigma} = c_{j,\sigma} e^{-\frac{g_j}{\hbar\omega_0}(a^\dagger - a)} \qquad \tilde{c}^\dagger_{j,\sigma} = c^\dagger_{j,\sigma} e^{+\frac{g_j}{\hbar\omega_0}(a^\dagger - a)} \tag{S40}$$

The transformed Hamiltonian is:

$$\begin{aligned}\tilde{H}_{\text{Hol}} = &\sum_{j=1}^{N}\sum_{\sigma=\uparrow,\downarrow} \epsilon_j\, \tilde{c}^\dagger_{j,\sigma}\tilde{c}_{j,\sigma} - \sum_{j=1}^{N-1}\sum_{\sigma}\left[t_j\, \tilde{c}^\dagger_{j\sigma}\tilde{c}_{j+1\sigma} - i\lambda_j\, \tilde{c}^\dagger_{j\sigma}\vec{v}_j\cdot\vec{\sigma}\tilde{c}_{j+1\sigma} + \text{h.c.}\right] \\ &+ \hbar\omega_0\left(\tilde{a}^\dagger\tilde{a} + \frac{1}{2}\right) + \sum_{j=1}^{2}\sum_{\sigma=\uparrow,\downarrow} g_j\, \tilde{c}^\dagger_{j,\sigma}\tilde{c}_{j,\sigma}\left(\tilde{a}^\dagger + \tilde{a}\right) + \text{h.c.}\end{aligned} \tag{S41}$$

We calculate:

$$\tilde{c}^\dagger_{j,\sigma}\tilde{c}_{j,\sigma} = c^\dagger_{j,\sigma} c_{j,\sigma}$$

$$\tilde{c}^\dagger_{j,\sigma}\tilde{c}_{j',\sigma} = c^\dagger_{j,\sigma} c_{j',\sigma}\, e^{\frac{g_j - g_{j'}}{\hbar\omega_0}(a^\dagger - a)}$$

$$\tilde{a}^\dagger + \tilde{a} = a^\dagger + a - 2\sum_{j=1}^{N}\sum_{\sigma=\uparrow,\downarrow} \frac{g_j}{\hbar\omega_0}\, c^\dagger_{j,\sigma} c_{j,\sigma}$$

$$\tilde{a}^\dagger\tilde{a} + \frac{1}{2} = a^\dagger a + \frac{1}{2} - \sum_{j=1}^{N}\sum_{\sigma=\uparrow,\downarrow} \frac{g_j}{\hbar\omega_0}\, c^\dagger_{j,\sigma} c_{j,\sigma}(a^\dagger + a) + \left[\sum_{j=1}^{N}\sum_{\sigma=\uparrow,\downarrow} \frac{g_j}{\hbar\omega_0}\, c^\dagger_{j,\sigma} c_{j,\sigma}\right]^2$$

First we evaluate:

$$\begin{aligned}&\sum_{j=1}^{N}\sum_{\sigma=\uparrow,\downarrow} \epsilon_j\, \tilde{c}^\dagger_{j,\sigma}\tilde{c}_{j,\sigma} + \hbar\omega_0\left(\tilde{a}^\dagger\tilde{a} + \frac{1}{2}\right) + \sum_{j=1}^{N}\sum_{\sigma=\uparrow,\downarrow} \frac{g_j}{\hbar\omega_0}\, \tilde{c}^\dagger_{j,\sigma}\tilde{c}_{j,\sigma}\left(\tilde{a}^\dagger + \tilde{a}\right) \\ &= \sum_{j=1}^{N}\sum_{\sigma=\uparrow,\downarrow} \epsilon_j\, c^\dagger_{j,\sigma} c_{j,\sigma} + \hbar\omega_0\left(a^\dagger a + \frac{1}{2}\right) - \frac{1}{\hbar\omega_0}\left[\sum_{j=1}^{N}\sum_{\sigma=\uparrow,\downarrow} g_j\, c^\dagger_{j,\sigma} c_{j,\sigma}\right]^2.\end{aligned}$$

Now we observe:

$$
\begin{aligned}
\left[\sum_{j=1}^{N}\sum_{\sigma=\uparrow,\downarrow} g_j\, c^\dagger_{j,\sigma}c_{j,\sigma}\right]^2 &= \left[\sum_{j=1}^{N} g_j^2 \sum_{\sigma=\uparrow,\downarrow} n_{j,\sigma}\right]^2\\
&= \left[\sum_{j=1}^{N} g_j^2\Big(n_{j,\uparrow}+n_{j,\downarrow}\Big)\right]^2\\
&= \sum_{j=1}^{N} g_j^2\Big(n_{j,\uparrow}+n_{j,\downarrow}\Big)\\
&= \sum_{j=1}^{N} g_j^2\, n_{j,\sigma}\,,
\end{aligned}
$$

where we have used the two facts:

- Fermi–Dirac statistics: $n^2_{j,\sigma} = n_{j,\sigma}$.
- Single Electron Theory: $n_{j,\sigma}n_{k,\sigma'} = 0$.

In the end we get:

$$
\begin{aligned}
\tilde{H}_{\text{Hol}} =& \sum_{j=1}^{N}\sum_{\sigma}\left[\epsilon_j - \frac{g_j^2}{\hbar\omega_0}\right] c^\dagger_{j\sigma}c_{j\sigma} + \hbar\omega_0\left(a^\dagger a + \frac{1}{2}\right)\\
&- \sum_{j=1}^{N-1}\sum_{\sigma}\left[t_j\, c^\dagger_{j\sigma}c_{j+1\sigma} - i\lambda_j\, c^\dagger_{j\sigma}\vec{v}_j\cdot\vec{\sigma}\, c_{j+1\sigma}\right] e^{\frac{g_j-g_{j+1}}{\hbar\omega_0}(a^\dagger - a)} + \text{h.c.}
\end{aligned}
\tag{S42}
$$

But at this point, since the fermionic and bosonic hilbert space are completely factorized we can find a gauge transformation to absorb the spin-dependent part by obtaining that even in this case there will be no polarization like we have done with (S1).

A. Multiple Mode

The single–mode derivation extends straightforwardly to the case of multiple independent Holstein modes. Starting from

$$
H^{(M)}_{\text{Hol}} = H_0 + \sum_{\mu=1}^{M}\hbar\omega_{0,\mu}\big(a^\dagger_\mu a_\mu + \tfrac{1}{2}\big) + \sum_{\mu=1}^{M}\sum_{j=1}^{N} g_{j,\mu}\,(a^\dagger_\mu + a_\mu)\sum_{\sigma} c^\dagger_{j\sigma}c_{j\sigma}, \tag{S43}
$$

one introduces the generalized Lang–Firsov generator

$$
\mathbf{S} = \sum_{\mu=1}^{M}\sum_{j=1}^{N}\sum_{\sigma}\frac{g_{j,\mu}}{\hbar\omega_{0,\mu}}\, c^\dagger_{j\sigma}c_{j\sigma}\,(a^\dagger_\mu - a_\mu), \tag{S44}
$$

which again satisfies $\mathbf{S}^\dagger = -\mathbf{S}$. Since different modes commute, $[\mathbf{S}_\mu, \mathbf{S}_{\mu'}] = 0$, the transformed Hamiltonian is simply obtained by applying each single–mode displacement in sequence. The resulting expression is the natural generalization of Eq. (S42), with the bosonic dressing factors acquiring a multimode contribution,

$$e^{\frac{g_{j,\mu}-g_{j+1,\mu}}{\hbar\omega_{0,\mu}}(a_\mu^\dagger - a_\mu)} \quad \longrightarrow \quad \exp\left[\sum_{\mu=1}^{M} \frac{g_{j,\mu}-g_{j+1,\mu}}{\hbar\omega_{0,\mu}}(a_\mu^\dagger - a_\mu)\right]. \tag{S45}$$

Crucially, the fermionic and bosonic sectors still factorize exactly, and the spin-dependent hopping retains the same SU(2) structure as in H_0. Thus, even in the presence of multiple Holstein vibrational modes, the spin dynamics can be gauged away and no spin polarization can arise in single-channel models.

IV. PEIERLS COUPLING AND THE BREAKING OF THE SPIN GAUGE

Now let's try to couple the fermionic Hamiltonian with Peierls-like bosons. For simplicity and clarity, let's start with the calculation using two sites on the chain.

$$\begin{aligned} H_{P,2} = &\sum_{j=1}^{2}\sum_{\sigma} \epsilon_j c_{j\sigma}^\dagger c_{j\sigma} + \hbar\omega_0\left(a^\dagger a + \frac{1}{2}\right) \\ &+ \sum_{\sigma} c_{1\sigma}^\dagger c_{2\sigma}\left[-t - \frac{g}{\sqrt{2}}(a^\dagger + a)\right] + \text{h.c.} \\ &+ i\sum_{\sigma} c_{1\sigma}^\dagger \vec{v}\cdot\vec{\sigma} c_{2\sigma}\left[\lambda + \frac{\chi}{\sqrt{2}}(a^\dagger + a)\right] + \text{h.c.}. \end{aligned} \tag{S46}$$

In matrix form, the only fermionic part is

$$H_f = \left[\begin{array}{cc|cc} \epsilon_1 & 0 & -t + i\lambda v_z & \lambda(iv_x + v_y) \\ 0 & \epsilon_1 & \lambda(iv_x - v_y) & -t - i\lambda v_z \\ \hline -t - i\lambda v_z & \lambda(-iv_x - v_y) & \epsilon_2 & 0 \\ \lambda(-iv_x + v_y) & -t + i\lambda v_z & 0 & \epsilon_2 \end{array}\right],$$

while the interaction part

$$H_{int} = \frac{1}{\sqrt{2}}\left[\begin{array}{cc|cc} 0 & 0 & -g + i\chi v_z & \chi(iv_x + v_y) \\ 0 & 0 & \chi(iv_x - v_y) & -g - i\chi v_z \\ \hline -g - i\chi v_z & \chi(-iv_x - v_y) & 0 & 0 \\ \chi(-iv_x + v_y) & -g + i\chi v_z & 0 & 0 \end{array}\right].$$

To use matrix language, we need to introduce

$$\mathbf{c} = \begin{bmatrix} c_{1,\uparrow} \\ \\ c_{1,\downarrow} \\ \\ c_{2,\uparrow} \\ \\ c_{2,\downarrow} \end{bmatrix} \qquad \mathbf{c}^\dagger = \begin{bmatrix} c^\dagger_{1,\uparrow} \ , \ c^\dagger_{1,\downarrow} \ , \ c^\dagger_{2,\uparrow} \ , \ c^\dagger_{2,\downarrow} \end{bmatrix}$$

and at this point we can rewrite the entire Hamiltonian as

$$H_{P,2} = \mathbf{c}^\dagger H_f \mathbf{c} + \hbar\omega_0 \Big(a^\dagger a + \frac{1}{2}\Big) + \mathbf{c}^\dagger H_{int} \mathbf{c}(a^\dagger + a). \tag{S47}$$

We now observe that

$$H_f = H_f^\dagger,$$

$$H_{int} = H_{int}^\dagger.$$

Since they are Hermitian square matrices of finite order (4×4) then they are Normal matrices and therefore the spectral theorem holds. So they are diagonalizable by a unit matrix and possess only real eigenvalues; in particular, eigenvectors related to distinct eigenvalues are orthogonal to each other. We apply this important result to H_{int}:

$$H_{int} = P Q_{int} P^\dagger. \tag{S48}$$

Where the matrix Q_{int} is diagonal (with eigenvalues q_j) and for the matrix P holds

$$PP^\dagger = \mathbf{1} = P^\dagger P. \tag{S49}$$

So we can write

$$\begin{aligned} H_{P,2} &= \mathbf{c}^\dagger P P^\dagger H_f P P^\dagger \mathbf{c} + \hbar\omega_0 \Big(a^\dagger a + \frac{1}{2}\Big) + \mathbf{c}^\dagger P P^\dagger H_{int} P P^\dagger \mathbf{c}(a^\dagger + a) \\ &= \mathbf{c}^\dagger P P^\dagger H_f P P^\dagger \mathbf{c} + \hbar\omega_0 \Big(a^\dagger a + \frac{1}{2}\Big) + \mathbf{c}^\dagger P Q_{int} P^\dagger \mathbf{c}(a^\dagger + a). \end{aligned} \tag{S50}$$

Now we transform $\mathbf{c}$ particles into $\mathbf{f}$ particles by defining

$$\mathbf{f}^\dagger = \mathbf{c}^\dagger P \qquad\qquad \mathbf{f} = P^\dagger \mathbf{c}. \tag{S51}$$

Before proceeding further, let us see what this transformation entails:

$$Q_{int} = \frac{\sqrt{g^2 + \chi^2 ||\vec{v}||^2}}{\sqrt{2}} \begin{pmatrix} -1 & 0 & 0 & 0 \\ 0 & -1 & 0 & 0 \\ 0 & 0 & +1 & 0 \\ 0 & 0 & 0 & +1 \end{pmatrix} \tag{S52}$$

$$P = \frac{1}{\sqrt{2}} \begin{pmatrix} \frac{\chi(-iv_x - v_y)}{\sqrt{g^2+\chi^2||\vec{v}||^2}} & \frac{+g - i\chi v_z}{\sqrt{g^2+\chi^2||\vec{v}||^2}} & \frac{\chi(+iv_x + v_y)}{\sqrt{g^2+\chi^2||\vec{v}||^2}} & \frac{-g + i\chi v_z}{\sqrt{g^2+\chi^2||\vec{v}||^2}} \\ \frac{+g + i\chi v_z}{\sqrt{g^2+\chi^2||\vec{v}||^2}} & \frac{\chi(-iv_x + v_y)}{\sqrt{g^2+\chi^2||\vec{v}||^2}} & \frac{-g - i\chi v_z}{\sqrt{g^2+\chi^2||\vec{v}||^2}} & \frac{\chi(+iv_x - v_y)}{\sqrt{g^2+\chi^2||\vec{v}||^2}} \\ 0 & 1 & 0 & 1 \\ 1 & 0 & 1 & 0 \end{pmatrix}. \tag{S53}$$

So the new $\mathbf{f}$ operators have the following form:

$$\mathbf{f} = P^\dagger \mathbf{c} = \frac{1}{\sqrt{2}} \begin{pmatrix} \frac{\chi\left(+iv_x - v_y\right)}{\sqrt{g^2 + \chi^2||\vec{v}||^2}} & \frac{g - i\chi v_z}{\sqrt{g^2 + \chi^2||\vec{v}||^2}} & 0 & 1 \\ \frac{g + i\chi v_z}{\sqrt{g^2 + \chi^2||\vec{v}||^2}} & \frac{\chi\left(+iv_x + v_y\right)}{\sqrt{g^2 + \chi^2||\vec{v}||^2}} & 1 & 0 \\ \frac{\chi\left(-iv_x + v_y\right)}{\sqrt{g^2 + \chi^2||\vec{v}||^2}} & \frac{-g + i\chi v_z}{\sqrt{g^2 + \chi^2||\vec{v}||^2}} & 0 & 1 \\ \frac{-g - i\chi v_z}{\sqrt{g^2 + \chi^2||\vec{v}||^2}} & \frac{\chi\left(-iv_x - v_y\right)}{\sqrt{g^2 + \chi^2||\vec{v}||^2}} & 1 & 0 \end{pmatrix} \begin{bmatrix} c_{1,\uparrow} \\ c_{1,\downarrow} \\ c_{2,\uparrow} \\ c_{2,\downarrow} \end{bmatrix}$$

$$= \frac{1}{\sqrt{2}} \begin{bmatrix} \frac{\chi(iv_x - v_y)}{\sqrt{g^2 + \chi^2||\vec{v}||^2}} c_{1,\uparrow} + \frac{g - i\chi v_z}{\sqrt{g^2 + \chi^2||\vec{v}||^2}} c_{1,\downarrow} + c_{2,\downarrow} \\ \frac{g + i\chi v_z}{\sqrt{g^2 + \chi^2||\vec{v}||^2}} c_{1,\uparrow} + \frac{\chi(iv_x + v_y)}{\sqrt{g^2 + \chi^2||\vec{v}||^2}} c_{1,\downarrow} + c_{2,\uparrow} \\ \frac{\chi(-iv_x + v_y)}{\sqrt{g^2 + \chi^2||\vec{v}||^2}} c_{1,\uparrow} + \frac{-g + i\chi v_z}{\sqrt{g^2 + \chi^2||\vec{v}||^2}} c_{1,\downarrow} + c_{2,\downarrow} \\ \frac{-g - i\chi v_z}{\sqrt{g^2 + \chi^2||\vec{v}||^2}} c_{1,\uparrow} + \frac{\chi(-iv_x - v_y)}{\sqrt{g^2 + \chi^2||\vec{v}||^2}} c_{1,\downarrow} + c_{2,\uparrow} \end{bmatrix} = \begin{bmatrix} f_{1,\uparrow} \\ f_{1,\downarrow} \\ f_{2,\uparrow} \\ f_{2,\downarrow} \end{bmatrix}$$

These new operators are linear combinations of the previous ones. Now we proceed to study the Hamiltonian and obtain

$$\begin{aligned} H_{P,2} &= \mathbf{c}^\dagger P P^\dagger H_f P P^\dagger \mathbf{c} + \hbar\omega_0\Big(a^\dagger a + \frac{1}{2}\Big) + \mathbf{c}^\dagger P Q_{int} P^\dagger \mathbf{c}(a^\dagger + a) \\ &= \mathbf{f}^\dagger P^\dagger H_f P \mathbf{f} + \hbar\omega_0\Big(a^\dagger a + \frac{1}{2}\Big) + \mathbf{f}^\dagger Q_{int} \mathbf{f}(a^\dagger + a) \\ &= \sum_{\alpha,\beta=1}^{4} (\hat{H}_f)_{\alpha,\beta} f^\dagger_\alpha f_\beta + \hbar\omega_0\Big(a^\dagger a + \frac{1}{2}\Big) + \sum_{\alpha=1}^{4} q_\alpha f^\dagger_\alpha f_\alpha (a^\dagger + a). \end{aligned} \tag{S54}$$

Where the q_α are the eigenvalues of the matrix H_{int} (which we recall to be real). We observe that the index j running on the molecular sites while the indices α and β refer to the matrices notation. Now we apply to the new Hamiltonian with $\mathbf{f}$ operators the Lang-Firsov Transformation and following the same procedure as seen in section III and we obtain

$$\tilde{H}_{P,2} = \sum_{\alpha,\beta=1}^{4} (\hat{H}_f)_{\alpha,\beta} f^\dagger_\alpha f_\beta e^{\frac{q_\alpha - q_\beta}{\hbar\omega_0}(a^\dagger - a)} + \hbar\omega_0\Big(a^\dagger a + \frac{1}{2}\Big) - \frac{1}{\hbar\omega_0}\sum_{\alpha=1}^{4} q_\alpha^2 f^\dagger_\alpha f_\alpha. \tag{S55}$$

We observe that in the new $\hat{H}_f$ matrix the spin structure is maintained so it has the form:

$$\hat{H}_f = \left[\begin{array}{cc|cc} \tilde{\epsilon}_1 & 0 & -\tilde{t} + i\tilde{\lambda}\tilde{v}_z & \tilde{\lambda}(i\tilde{v}_x + \tilde{v}_y) \\ 0 & \tilde{\epsilon}_1 & \tilde{\lambda}(i\tilde{v}_x - \tilde{v}_y) & -\tilde{t} - i\tilde{\lambda}\tilde{v}_z \\ \hline -\tilde{t} - i\tilde{\lambda}\tilde{v}_z & \tilde{\lambda}(-i\tilde{v}_x - \tilde{v}_y) & \tilde{\epsilon}_2 & 0 \\ \tilde{\lambda}(-i\tilde{v}_x + \tilde{v}_y) & -\tilde{t} + i\tilde{\lambda}v_z & 0 & \tilde{\epsilon}_2 \end{array}\right],$$

where these new parameters depend on the previous ones and the matrix P. We can relate the new constants with the tilde to the starting constants:

$$\tilde{\epsilon}_1 = \frac{\epsilon_1 + \epsilon_2}{2} - \frac{gt + \chi\lambda||\vec{v}||^2}{\sqrt{g^2 + \chi^2||\vec{v}||^2}}$$

$$\tilde{\epsilon}_2 = \frac{\epsilon_1 + \epsilon_2}{2} + \frac{gt + \chi\lambda||\vec{v}||^2}{\sqrt{g^2 + \chi^2||\vec{v}||^2}}$$

$$\tilde{t} = \frac{\epsilon_1 - \epsilon_2}{2}$$

$$\tilde{\lambda}\tilde{v}_z = \frac{-(\lambda g - \chi t)v_z}{\sqrt{g^2 + \chi^2||\vec{v}||^2}} \qquad \tilde{\lambda}\tilde{v}_x = \frac{(\lambda g - \chi t)v_x}{\sqrt{g^2 + \chi^2||\vec{v}||^2}} \qquad \tilde{\lambda}\tilde{v}_y = \frac{-(\lambda g - \chi t)v_y}{\sqrt{g^2 + \chi^2||\vec{v}||^2}}$$

Now we focus only on the first term of (S55), which is the one that can give polarization in the base f. We can write it in the tight-binding formalism by observing that the spin

structure has been retained,

$$\sum_{\alpha,\beta=1}^{4}(\hat{H}_f)_{\alpha,\beta}f^\dagger_\alpha f_\beta e^{\frac{q_\alpha-q_\beta}{\hbar\omega_0}(a^\dagger-a)} = \sum_{j=1}^{2}\sum_\sigma \tilde{\epsilon}_j f^\dagger_{j\sigma}f_{j\sigma} - \sum_\sigma\left[\tilde{t}_j\, f^\dagger_{1\sigma}f_{2\sigma} - i\tilde{\lambda}\, f^\dagger_{1\sigma}\tilde{\vec{v}}\cdot\vec{\sigma}f_{2\sigma}\right]e^{-\frac{\sqrt{2(g^2+\chi^2||\vec{v}||^2)}}{\hbar\omega_0}(a^\dagger-a)} + \text{h.c.}. \tag{S56}$$

The final Hamiltonian is

$$\tilde{H}_{P,2} = \sum_{j=1}^{2}\sum_\sigma\left[\tilde{\epsilon}_j - \frac{g^2+\chi^2||\vec{v}||^2}{2\hbar\omega_0}\right]f^\dagger_{j\sigma}f_{j\sigma} + \hbar\omega_0\Big(a^\dagger a+\frac{1}{2}\Big) - \sum_\sigma\left[\tilde{t}_j\, f^\dagger_{1\sigma}f_{2\sigma} - i\tilde{\lambda}\, f^\dagger_{1\sigma}\tilde{\vec{v}}\cdot\vec{\sigma}f_{2\sigma}\right]e^{-\frac{\sqrt{2(g^2+\chi^2||\vec{v}||^2)}}{\hbar\omega_0}(a^\dagger-a)} + \text{h.c.}. \tag{S57}$$

Following the same procedure seen in section I,

$$\tilde{T} \equiv -\tilde{t}\mathbf{1}_{2\times2} + i\tilde{\lambda}\tilde{\vec{v}}\cdot\vec{\sigma} = -\tilde{t}\mathbf{1}_{2\times2} + i\tilde{\Lambda}\hat{n}\cdot\vec{\sigma} \quad \textit{with} \quad \tilde{\Lambda}\equiv\tilde{\lambda}||\tilde{\vec{v}}||,\ \hat{n}\equiv\frac{\tilde{\vec{v}}}{||\tilde{\vec{v}}||}. \tag{S58}$$

Now we use again identity (S6) and we define

$$\tilde{R}\equiv\sqrt{\tilde{t}^2+\tilde{\Lambda}^2}, \qquad \tilde{\phi}\equiv\arctan\Big(\tilde{\Lambda}/\tilde{t}\Big). \tag{S59}$$

it follows immediately that

$$\tilde{T} = -\tilde{R}\tilde{U} = -\tilde{R}\,e^{-i\tilde{\phi}\hat{n}\cdot\vec{\sigma}}\,, \tag{S60}$$

Now we define recursively the transformation as in (S10) and in the same way as in section I we obtain

$$f^\dagger_{1,\sigma}\tilde{T}f_{2,\sigma} = -\tilde{R}d^\dagger_{1,\sigma}d_{2,\sigma}\,. \tag{S61}$$

So in the end we get

$$\tilde{H}_{P,2} = \sum_{j=1}^{2}\sum_\sigma\left[\tilde{\epsilon}_j - \frac{g^2+\chi^2||\vec{v}||^2}{2\hbar\omega_0}\right]d^\dagger_{j,\sigma}d_{j,\sigma} - \tilde{R}\sum_\sigma\big(d^\dagger_{1,\sigma}d_{2,\sigma}\big)e^{-\frac{\sqrt{2(g^2+\chi^2||\vec{v}||^2)}}{\hbar\omega_0}(a^\dagger-a)} + \text{h.c.}. \tag{S62}$$

Here it would seem that the Hamiltonian (S62) cannot be polarized since it is always possible to find a transformation (S10). In this case there is a fundamental difference, the transformation used however unitary it is not a gauge transformation. We can show it:

$$d_{1,\sigma} = \mathbf{1}_{2\times2}f_{1,\sigma} = \mathbf{1}_{2\times2}\begin{bmatrix} f_{1,\uparrow} \\ f_{1,\downarrow}\end{bmatrix} = \frac{1}{\sqrt{2}}\begin{bmatrix} \frac{\chi(iv_x-v_y)}{\sqrt{g^2+\chi^2||\vec{v}||^2}}c_{1,\uparrow} + \frac{g-i\chi v_z}{\sqrt{g^2+\chi^2||\vec{v}||^2}}c_{1,\downarrow} + c_{2,\downarrow} \\ \frac{g+i\chi v_z}{\sqrt{g^2+\chi^2||\vec{v}||^2}}c_{1,\uparrow} + \frac{\chi(iv_x+v_y)}{\sqrt{g^2+\chi^2||\vec{v}||^2}}c_{1,\downarrow} + c_{2,\uparrow}\end{bmatrix}$$

$$d_{2,\sigma} = \tilde{U}^\dagger f_{2,\sigma} = e^{-i\tilde{\phi}\hat{n}\cdot\vec{\sigma}} \begin{bmatrix} f_{2,\uparrow} \\ \\ f_{2,\downarrow} \end{bmatrix}$$

$$= \frac{1}{\sqrt{2}} \begin{bmatrix} \cos\tilde{\phi} - i\,n_z\,\sin\tilde{\phi} & (-i\,n_x - n_y)\,\sin\tilde{\phi} \\ (-i\,n_x + n_y)\,\sin\tilde{\phi} & \cos\tilde{\phi} + i\,n_z\,\sin\tilde{\phi} \end{bmatrix} \begin{bmatrix} \frac{\chi(-iv_x + v_y)}{\sqrt{g^2+\chi^2||\vec{v}||^2}} c_{1,\uparrow} + \frac{-g + i\chi v_z}{\sqrt{g^2+\chi^2||\vec{v}||^2}} c_{1,\downarrow} + c_{2,\downarrow} \\ \frac{-g - i\chi v_z}{\sqrt{g^2+\chi^2||\vec{v}||^2}} c_{1,\uparrow} + \frac{\chi(-iv_x - v_y)}{\sqrt{g^2+\chi^2||\vec{v}||^2}} c_{1,\downarrow} + c_{2,\uparrow} \end{bmatrix}$$

We can clearly see that the gauge transformation acting on site 1 in the f basis gives different coefficients to the fermionic creation and annihilation operators in the c basis compared to the one acting on site 2 in the f basis. This makes the transformation non-local because a gauge transformation acts locally in the same way on an operator.

Equation (S56) shows that the transformed Hamiltonian becomes spin independent when the effective SOC term vanishes, *i.e.*,

$$\tilde{\lambda}\tilde{v}_x = \tilde{\lambda}\tilde{v}_y = \tilde{\lambda}\tilde{v}_z = 0\ ,\ \ i.e. \tag{S63}$$

$$\frac{g}{t} = \frac{\chi}{\lambda}. \tag{S64}$$

When the phonon-induced modulations are proportional for the spin-independent and spin-dependent parts of the bond operator. On this *symmetry-restoring* manifold the bond operator factorizes as a scalar (phonon-dependent) prefactor times a fixed SU(2) matrix, so that a site-local spin gauge can again be defined and the model maps onto a spin-independent form. When the condition $\frac{g}{t} = \frac{\chi}{\lambda}$ is not met, the gauge transformation is not possible allowing spin polarization already in the minimal single-channel setting.

V. WILSON-LOOP INTERPRETATION WITH PEIERLS BOSONS

We now derive the vibronic analogue of the Wilson loop introduced for the fermionic ring, starting from the Peierls–SOC dimer Hamiltonian

$$H_{P,2} = \sum_\sigma \left[c^\dagger_{1\sigma} T(X) c_{2\sigma} + c^\dagger_{2\sigma} T^\dagger(X) c_{1\sigma}\right] + \hbar\omega_0 \left(a^\dagger a + \frac{1}{2}\right), \tag{S65}$$

where the electron–phonon–spin–orbit coupling appears in the operator

$$T(X) = -[t + gX]\,\mathbf{1}_{2\times 2} + i[\lambda + \chi X]\,\vec{v}\cdot\vec{\sigma}, \qquad X = \frac{a^\dagger + a}{\sqrt{2}}. \tag{S66}$$

We identify the electronic–vibrational basis states

$$|A_0\rangle = |1\rangle_e \otimes |0\rangle_b, \qquad |B_0\rangle = |2\rangle_e \otimes |0\rangle_b,$$

and construct two coherent transport paths connecting them:

(i) **Direct electronic path:** $|1,0\rangle \to |2,0\rangle$, mediated by the purely electronic part of $T(X)$,

$$T_0 \equiv -t\,\mathbf{1} + i\lambda\,\vec{v}\cdot\vec{\sigma}. \tag{S67}$$

(ii) **Phonon–assisted path:** $|1,0\rangle \to |2,1\rangle \to |1,1\rangle \to |2,0\rangle$, mediated by emission and reabsorption of one phonon, described by

$$G \equiv -g\,\mathbf{1} + i\chi\,\vec{v}\cdot\vec{\sigma}. \tag{S68}$$

The two corresponding amplitudes are

$$\mathcal{A}_{\rm dir} = T_0, \tag{S69}$$

$$\mathcal{A}_{\rm bos} = G\,T_0^{-1}\,G. \tag{S70}$$

All spin operators can be expressed as linear combinations of Pauli matrices along the direction $\hat{n} = \vec{v}/\|\vec{v}\|$. Using the identity

$$(\hat{n}\cdot\vec{\sigma})^2 = \mathbf{1},$$

one can explicitly evaluate the product $G\,T_0^{-1}\,G$ as follows.

A. Explicit evaluation of the phonon–assisted amplitude

Let us write

$$T_0 = -t\,\mathbf{1} + i\lambda\,\|\vec{v}\|\,(\hat{n}\cdot\vec{\sigma}), \tag{S71}$$

$$T_0^{-1} = \frac{1}{\sqrt{t^2 + \lambda\|\vec{v}\|}}\left(-t\,\mathbf{1} - i\lambda\,\|\vec{v}\|\,(\hat{n}\cdot\vec{\sigma})\right), \tag{S72}$$

$$G = -g\,\mathbf{1} + i\chi\,\|\vec{v}\|\,(\hat{n}\cdot\vec{\sigma}). \tag{S73}$$

We compute:

$$\mathcal{A}_{\rm bos} = \frac{1}{\sqrt{t^2+\lambda^2\|\vec{v}\|^2}}(-g\,\mathbf{1} + i\chi\,\|\vec{v}\|\hat{n}\cdot\vec{\sigma})(-t\,\mathbf{1} - i\lambda\,\|\vec{v}\|\hat{n}\cdot\vec{\sigma})(-g\,\mathbf{1} + i\chi\,\|\vec{v}\|\hat{n}\cdot\vec{\sigma}). \tag{S74}$$

Expanding the first two factors:

$$(-g\,\mathbf{1} + i\chi\,\|\vec{v}\|\hat{n}\cdot\vec{\sigma})(-t\,\mathbf{1} - i\lambda\,\|\vec{v}\|\hat{n}\cdot\vec{\sigma}) = gt\,\mathbf{1} + i(g\lambda - t\chi)\,\|\vec{v}\|\hat{n}\cdot\vec{\sigma} + \chi\lambda\,\|\vec{v}\|^2(\hat{n}\cdot\vec{\sigma})^2 \tag{S75}$$

$$= (gt + \chi\lambda\|\vec{v}\|^2)\mathbf{1} + i(g\lambda - t\chi)\|\vec{v}\|\,\hat{n}\cdot\vec{\sigma}, \tag{S76}$$

since $(\hat{n}\cdot\vec{\sigma})^2 = \mathbf{1}$. Multiplying by the third factor $(-g\,\mathbf{1} + i\chi\,\|\vec{v}\|\hat{n}\cdot\vec{\sigma})$:

$$\mathcal{A}_{\rm bos} \propto \Big[(gt + \chi\lambda\|\vec{v}\|^2)\mathbf{1} + i(g\lambda - t\chi)\|\vec{v}\|\,\hat{n}\cdot\vec{\sigma}\Big](-g\,\mathbf{1} + i\chi\,\|\vec{v}\|\hat{n}\cdot\vec{\sigma})$$

$$= -g(gt + \chi\lambda\|\vec{v}\|^2)\mathbf{1} + i\chi(gt + \chi\lambda\|\vec{v}\|^2)\,\|\vec{v}\|\hat{n}\cdot\vec{\sigma} - ig(g\lambda - t\chi)\|\vec{v}\|\,\hat{n}\cdot\vec{\sigma} - \chi(g\lambda - t\chi)\|\vec{v}\|^2\mathbf{1}. \tag{S77}$$

Collecting real and imaginary coefficients:

$$\mathcal{A}_{\rm bos} \propto \big[-g(gt+\chi\lambda\|\vec{v}\|^2) - \chi(g\lambda - t\chi)\|\vec{v}\|^2\big]\mathbf{1} + i\big[\chi(gt+\chi\lambda\|\vec{v}\|^2) - g(g\lambda - t\chi)\big]\,\|\vec{v}\|\hat{n}\cdot\vec{\sigma}. \tag{S78}$$

Simplifying the coefficients:

$$\alpha = -g^2 t - 2g\chi\lambda\|\vec{v}\|^2 + \chi^2 t\|\vec{v}\|^2, \tag{S79}$$

$$\beta = 2\chi g t\|\vec{v}\| + (\chi^2\|\vec{v}\|^2 - g^2)\lambda\|\vec{v}\|. \tag{S80}$$

Thus,

$$\mathcal{A}_{\rm bos} = \frac{1}{\sqrt{t^2+\lambda^2\|\vec{v}\|^2}}\left[\alpha\,\mathbf{1} + i\beta\,\hat{n}\cdot\vec{\sigma}\right] = R_{\rm dir}\left[\alpha\,\mathbf{1} + i\beta\,\hat{n}\cdot\vec{\sigma}\right]. \tag{S81}$$

This matrix can again be decomposed into a modulus and an SU(2) rotation:

$$R_{\rm bos} = \sqrt{\alpha^2+\beta^2}, \qquad U_{\rm bos} = \frac{1}{R_{\rm bos}R_{\rm dir}}\mathcal{A}_{\rm bos} = e^{+i\phi_{\rm bos}\,\hat{n}\cdot\vec{\sigma}}, \tag{S82}$$

with

$$\tan\left(\phi_{\rm bos}\right) = \frac{\beta}{\alpha}. \tag{S83}$$

B. Direct amplitude

Similarly,

$$\mathcal{A}_{\rm dir} = -t\,\mathbf{1} + i\lambda\|\vec{v}\|\hat{n}\cdot\vec{\sigma} = -R_{\rm dir}e^{-i\phi_{\rm dir}\hat{n}\cdot\vec{\sigma}}, \tag{S84}$$

where

$$R_{\rm dir} = \sqrt{t^2+\lambda^2\|\vec{v}\|^2}, \qquad \tan(\phi_{\rm dir}) = \frac{\lambda\|\vec{v}\|}{t}. \tag{S85}$$

C. Vibronic Wilson loop

The relative SU(2) rotation between the two paths is defined as

$$\mathcal{W}_{\mathrm{vib}} = U_{\mathrm{dir}}^{\dagger} U_{\mathrm{bos}} = e^{+i\Phi_{\mathrm{vib}}\hat{n}\cdot\vec{\sigma}}, \qquad \Phi_{\mathrm{vib}} = \phi_{\mathrm{bos}} + \phi_{\mathrm{dir}}. \tag{S86}$$

This object represents the holonomy associated with a closed trajectory in the combined electronic–vibrational configuration space. It plays the role of a *vibronic Wilson loop*: if $\mathcal{W}_{\mathrm{vib}} = \mathbf{1}$, the two propagation paths are equivalent up to a global SU(2) phase, while if $\mathcal{W}_{\mathrm{vib}} \neq \mathbf{1}$, a non–trivial spin rotation is accumulated.

D. Interference between vibronic paths

The total amplitude from $|A_0\rangle$ to $|B_0\rangle$ can be written as

$$\mathcal{A}_{\mathrm{tot}} = \mathcal{A}_{\mathrm{dir}} + \mathcal{A}_{\mathrm{bos}} = -R_{\mathrm{dir}}\, U_{\mathrm{dir}}[\mathbf{1} + r\,\mathcal{W}_{\mathrm{vib}}]\,, \qquad r = -R_{\mathrm{bos}}. \tag{S87}$$

Averaging over spin degrees of freedom yields the total probability amplitude

$$\begin{aligned} \left\langle \|\mathcal{A}_{\mathrm{tot}}\|^2 \right\rangle_{\mathrm{spin}} &= \frac{R_{\mathrm{dir}}}{2}\mathrm{Tr}\left[(\mathbf{1} + r\,\mathcal{W}_{\mathrm{vib}})^{\dagger}(\mathbf{1} + r\,\mathcal{W}_{\mathrm{vib}})\right] \\ &= R_{\mathrm{dir}} + R_{\mathrm{dir}}R_{\mathrm{bos}}^2 - R_{\mathrm{dir}}R_{\mathrm{bos}}\frac{1}{2}\mathrm{Tr}\left[\mathcal{W}_{\mathrm{vib}}^{\dagger} + \mathcal{W}_{\mathrm{vib}}\right]. \end{aligned} \tag{S88}$$

Hence the spin–averaged interference term reads

$$I_{\mathrm{int}} = -2R_{\mathrm{dir}}R_{\mathrm{bos}}\cos(\Phi_{\mathrm{vib}}) = -2R_{\mathrm{dir}}R_{\mathrm{bos}}\cos(\phi_{\mathrm{bos}} + \phi_{\mathrm{dir}}). \tag{S89}$$

E. Condition for trivial vibronic loop

The vibronic Wilson loop becomes trivial when the two SU(2) phases coincide,

$$\Phi_{\mathrm{vib}} = 2\pi m \quad \text{with} \quad m \in \mathrm{N} \tag{S90}$$

$$2\pi m = \Phi_{\mathrm{vib}} = \phi_{\mathrm{bos}} + \phi_{\mathrm{dir}} = \arctan\left(\frac{\beta}{\alpha}\right) + \arctan\left(\frac{\lambda\|\vec{v}\|}{t}\right), \tag{S91}$$

but the arctan function can only range from $-\pi/2$ to $+\pi/2$, so the only possible solution is for $m = 0$ so

$$-\frac{\beta}{\alpha} = \frac{\lambda\|\vec{v}\|}{t} \quad \Longleftrightarrow \quad \frac{g}{t} = \frac{\chi}{\lambda}. \tag{S92}$$

Under this condition ($\frac{g}{t} = \frac{\chi}{\lambda} = \kappa$), all hopping operators $T(X)$ share the same spin–rotation axis and angle,

$$\begin{aligned} T(X) &= -[t + gX]\,\mathbf{1}_{2\times2} + i[\lambda + \chi X]\,\vec{v}\cdot\vec{\sigma} \\ &= -t\,[1 + \kappa X]\;\mathbf{1}_{2\times2} + i\lambda\,[1 + \kappa X]\;\vec{v}\cdot\vec{\sigma} \\ &= [1 + \kappa X]\,(-t\,\mathbf{1}_{2\times2} + i\lambda\,\vec{v}\cdot\vec{\sigma}) \\ &= f(X)\,T_0, \qquad f(X) \in \mathbb{R}, \end{aligned} \tag{S93}$$

and the spin dynamics factorizes from the vibrational degrees of freedom. Consequently, $\mathcal{W}_{\text{vib}} = \mathbf{1}$ for any phonon configuration, and the system cannot exhibit polarization even in the strong coupling regime.

This factorization property is an operator identity on the full vibronic Hilbert space: the dependence on the phonon coordinate X enters only through the real scalar prefactor $f(X)$, while the SU(2) structure is entirely fixed by the X–independent matrix T_0. Therefore, any multi–phonon process composed of an arbitrary sequence of hoppings $T(X_1), T(X_2), \ldots, T(X_{N_b})$ reduces to

$$T(X_{N_b})\cdots T(X_2)T(X_1) = \big[f(X_{N_b})\cdots f(X_2)f(X_1)\big]\cdot T_0^{N_b}, \tag{S94}$$

so that all vibronic paths share the same SU(2) rotation. As a consequence, the vibronic Wilson loop remains trivial for any number of phonons N_b, and no spin polarization can emerge even in the presence of arbitrarily high vibronic excitations.

VI. EXTENSION OF THE VIBRONIC WILSON-LOOP ANALYSIS TO ARBITRARY CHAINS AND MULTIPLE MODES

The vibronic WL analysis discussed in the main text is not specific to the two-site toy model, but extends naturally to longer chains and to multiple vibratinal modes. We first consider an N-site chain with bond-dependent parameters coupled to a single Peierls-active mode,

$$\begin{aligned} H_{P,N} &= \sum_{j=1}^{N}\sum_{\sigma} \epsilon_j c_{j\sigma}^{\dagger} c_{j\sigma} + \hbar\omega_0\big(a^{\dagger}a + \tfrac{1}{2}\big) \\ &+ \sum_{j=1}^{N-1}\sum_{\sigma} c_{j\sigma}^{\dagger} c_{j+1\sigma}\left[-t_j - \frac{g_j}{\sqrt{2}}(a^{\dagger} + a)\right] + \text{h.c.} \\ &+ i\sum_{j=1}^{N-1}\sum_{\sigma} c_{j\sigma}^{\dagger}(\vec{v}_j\cdot\vec{\sigma})\,c_{j+1\sigma}\left[\lambda_j + \frac{\chi_j}{\sqrt{2}}(a^{\dagger} + a)\right] + \text{h.c.}\,, \end{aligned} \tag{S95}$$

In principle, one could follow the same diagonalization-shift-reduction route as in Sec. **??**, but the interaction matrix now becomes $2N \times 2N$ and a closed-form diagonalization is generally cumbersome. Importantly, however, the qualitative outcome can be established directly from the WL, without performing this explicit construction. The key object is again the Peierls-dressed bond operator. On each bond j it reads

$$T_j(X) = -[t_j + g_j X]\,\mathbf{1} + i[\lambda_j + \chi_j X]\,\vec{v}_j\cdot\vec{\sigma}, \qquad X = \frac{a^\dagger + a}{\sqrt{2}}. \tag{S96}$$

The triviality of all elementary vibronic WLs requires the X-dependence of each bond operator to factorize into a *real scalar* prefactor multiplying a fixed SU(2) matrix, *i.e.* $T_j(X) = f_j(X)\,T_{0,j}$ with $f_j(X) \in \mathbb{R}$. This factorization holds if and only if the phonon-induced modulations are proportional for the spin-independent and spin-dependent parts,

$$\frac{g_j}{t_j} = \frac{\chi_j}{\lambda_j} \qquad \forall\, j. \tag{S97}$$

On this bondwise symmetry-restoring manifold, all vibronic trajectories across the chain acquire the same SU(2) rotation given by the ordered product $T_{0,N-1}\cdots T_{0,2}T_{0,1}$. Equivalently, all vibronic WLs factorize into products of trivial bondwise loops and therefore remain trivial, so no spin polarization can be generated. Conversely, any deviation from (S97) produces a non-trivial vibronic WL, breaks the site-local spin gauge, and enables spin polarization.

In realistic molecular systems, several Peierls-active modes may couple to the same bond. For an N-site chain with multiple Peierls modes denoted by the index μ,

$$\begin{aligned} H_{P,N}^{(\text{multi})} &= H_0 + \sum_{\mu} \hbar\omega_{0,\mu}\Big(a_\mu^\dagger a_\mu + \tfrac{1}{2}\Big) \\ &+ \sum_{\mu}\sum_{j=1}^{N-1}\sum_{\sigma}\Big[-g_{j,\mu} + i\chi_{j,\mu}\,\vec{v}_j\cdot\vec{\sigma}\Big](a_\mu^\dagger + a_\mu)\,c_{j\sigma}^\dagger c_{j+1\sigma} + \text{h.c.}, \end{aligned} \tag{S98}$$

a closed-form analogue of the two-site diagonalization is not available in general. Nevertheless, the same factorization argument applies mode by mode: each Peierls contribution can be written as

$$T_{j,\mu}(X_\mu) = -[t_j + g_{j,\mu}X_\mu]\,\mathbf{1} + i[\lambda_j + \chi_{j,\mu}X_\mu]\,\vec{v}_j\cdot\vec{\sigma}, \tag{S99}$$

and factorizes as $T_{j,\mu}(X_\mu) = f_{j,\mu}(X_\mu)\,T_{0,j}$ with $f_{j,\mu}(X_\mu) \in \mathbb{R}$ if and only if

$$\frac{g_{j,\mu}}{t_j} = \frac{\chi_{j,\mu}}{\lambda_j} \qquad \forall\, j,\mu. \tag{S100}$$

In summary, Peierls couplings act directly on bond operators and generically yield non-trivial vibronic WLs, thereby breaking the site-local SU(2) spin gauge that makes single-channel electronic models spin independent. This establishes Peierl modes as a minimal route to enable CISS in open single-channel tight binding models.

VII. DERIVATION OF THE WL IN COHERENT DYNAMICS

We consider a single-particle tight-binding description on a discrete set of sites $\{|i\rangle\}_{i=1,\ldots,N}$ and a spin-$\frac{1}{2}$ internal degree of freedom. The Hilbert space factorizes as

$$\mathcal{H} = \mathcal{H}_{\mathrm{site}} \otimes \mathcal{H}_{\mathrm{spin}}, \qquad \dim \mathcal{H}_{\mathrm{spin}} = 2. \tag{S101}$$

We denote by $\mathbf{1}$ the identity operator in spin space.

a. Tight-binding Hamiltonian. We write the total Hamiltonian $\hat{H}$ as an on-site part plus spin-dependent hoppings,

$$\hat{H} = \hat{H}_{\mathrm{onsite}} + \hat{H}_{\mathrm{hop}}, \tag{S102}$$

with

$$\hat{H}_{\mathrm{onsite}} = \sum_i |i\rangle\langle i| \otimes \varepsilon_i \, \mathbf{1}, \tag{S103}$$

and

$$\hat{H}_{\mathrm{hop}} = \sum_{i \neq j} |j\rangle\langle i| \otimes T_{ji}. \tag{S104}$$

Here T_{ji} is a 2×2 matrix acting on $\mathcal{H}_{\mathrm{spin}}$, encoding the spin structure of the hopping process from site i to site j. Hermiticity implies $T_{ij} = T_{ji}^{\dagger}$.

For the class of tight-binding models considered in this work, each hopping matrix admits a polar decomposition into a scalar amplitude and an SU(2) rotation,

$$T_{ji} = R_{ji} \, U_{ji}, \qquad R_{ji} \in \mathbb{R}, \quad U_{ji} \in SU(2), \tag{S105}$$

where U_{ji} represents the spin rotation associated with the link $i \to j$.

As a concrete example (see Main), for nearest-neighbour hopping with spin–orbit coupling one has

$$T_j \equiv T_{j+1,j} = -t_j \, \mathbf{1} + i\lambda_j \, \hat{\mathbf{n}}_j \cdot \boldsymbol{\sigma} = -R_j \, e^{-i\varphi_j \, \hat{\mathbf{n}}_j \cdot \boldsymbol{\sigma}}, \tag{S106}$$

with $R_j = \sqrt{t_j^2 + \lambda_j^2}$ and $\tan\varphi_j = \lambda_j / t_j$.

b. Unitary time evolution: power-series expansion. The unitary time-evolution operator on the full Hilbert space is

$$\hat{U}(t) = e^{-i\hat{H}t}. \tag{S107}$$

Expanding in a power series yields

$$\hat{U}(t) = \sum_{n=0}^{\infty} \frac{(-it)^n}{n!} \hat{H}^n. \tag{S108}$$

We will use Eq. (S108) to express matrix elements of $\hat{U}(t)$ as a coherent sum over lattice paths, by inserting resolutions of the identity in $\mathcal{H}_{\text{site}}$ between successive factors of $\hat{H}$.
We define the spin-space propagator (a 2×2 matrix) between sites i and j as the site-projected matrix element of the full unitary operator,

$$\mathcal{U}_{ji}(t) \;\equiv\; \langle j|\hat{U}(t)|i\rangle, \tag{S109}$$

so that $\mathcal{U}_{ji}(t)$ acts only on $\mathcal{H}_{\text{spin}}$.
Using the series expansion (S108), we obtain

$$\mathcal{U}_{ji}(t) = \sum_{n=0}^{\infty} \frac{(-it)^n}{n!} \langle j|\hat{H}^n|i\rangle. \tag{S110}$$

To rewrite $\langle j|\hat{H}^n|i\rangle$ as a sum over sequences of intermediate sites, we insert $n-1$ identity in $\mathcal{H}_{\text{site}}$,

$$\mathbf{1}_{\text{site}} = \sum_k |k\rangle\langle k|. \tag{S111}$$

This yields, for $n \geq 1$,

$$\begin{aligned} \langle j|\hat{H}^n|i\rangle &= \sum_{k_{n-1},\ldots,k_1} \langle j|\hat{H}|k_{n-1}\rangle\langle k_{n-1}|\hat{H}|k_{n-2}\rangle \cdots \langle k_1|\hat{H}|i\rangle \\ &= \sum_{k_{n-1},\ldots,k_1} H_{jk_{n-1}}\, H_{k_{n-1}k_{n-2}} \,\cdots\, H_{k_1 i}, \end{aligned} \tag{S112}$$

where we introduced the spin-space blocks of the Hamiltonian,

$$H_{ji} \equiv \langle j|\hat{H}|i\rangle, \tag{S113}$$

which act on $\mathcal{H}_{\text{spin}}$.

c. Discrete paths. Equation (S112) has a natural path interpretation: each choice of $(k_1, \dots, k_{n-1})$ defines a discrete path

$$p: \qquad i = k_0 \to k_1 \to k_2 \to \cdots \to k_{n-1} \to k_n = j, \tag{S114}$$

of length n, and contributes the ordered product of matrices $H_{k_{\ell+1}k_\ell}$ along that path. Substituting Eq. (S112) into Eq. (S110), we obtain the explicit sum over paths,

$$\mathcal{U}_{ji}(t) = \delta_{ji}\,\mathbf{1} + \sum_{n=1}^{\infty} \frac{(-it)^n}{n!} \sum_{k_{n-1},\dots,k_1} H_{jk_{n-1}}\, H_{k_{n-1}k_{n-2}} \cdots H_{k_1 i}. \tag{S115}$$

In the next step, we use the tight-binding structure (S103)–(S104) (and in particular the factorization (S105) of the hopping matrices) to separate, for each path, a scalar amplitude from an ordered SU(2) spin rotation.

From Eqs. (S103)–(S104), the site-projected blocks of the Hamiltonian read

$$H_{ji} = \langle j|\hat{H}|i\rangle = \delta_{ji}\,\varepsilon_i\,\mathbf{1} + (1-\delta_{ji})\,T_{ji}. \tag{S116}$$

In particular, whenever the step $i \to j$ corresponds to a hopping process, we may write $T_{ji} = R_{ji}U_{ji}$ with $R_{ji} \in \mathbb{R}$ and $U_{ji} \in SU(2)$ (Eq. (S105)).

To keep the notation compact, it is convenient to focus on paths made of hopping steps (the generalization including on-site "waiting" steps is straightforward and only modifies scalar prefactors). For a length-n hopping path $p: i = k_0 \to k_1 \to \cdots \to k_n = j$, Eq. (S112) gives the ordered product

$$M_p \equiv T_{k_n k_{n-1}}\, T_{k_{n-1}k_{n-2}} \cdots T_{k_1 k_0}. \tag{S117}$$

Using $T_{ji} = R_{ji}U_{ji}$, we can factorize

$$M_p = \Big(\prod_{\ell=0}^{n-1} R_{k_{\ell+1}k_\ell}\Big)\Big(\prod_{\ell=0}^{n-1} U_{k_{\ell+1}k_\ell}\Big) \equiv a_p\, U_p, \tag{S118}$$

where a_p is a scalar amplitude and

$$U_p \equiv \prod_{\ell=0}^{n-1} U_{k_{\ell+1}k_\ell} \in SU(2)\,. \tag{S119}$$

Here the single index p labels the full path connecting i to j, and U_p denotes the corresponding path-ordered SU(2) product of link matrices, i.e. the total spin rotation associated with that specific trajectory.

d. Coherent sum over paths. Collecting the contributions of all paths from i to j at all lengths, we can write the site-to-site propagator (S109) as

$$\mathcal{U}_{ji}(t) = \sum_{p:i\to j} A_p(t)\, U_p, \tag{S120}$$

where p runs over all (discrete) paths connecting i to j and the scalar weights $A_p(t)$ encode both the time dependence (from the series in t) and the spin-independent amplitudes associated with each path. More explicitly, $A_p(t)$ collects the factor $(-it)^n/n!$ and the scalar product $\prod_\ell R_{k_{\ell+1}k_\ell}$ for paths of length n, and, if included, additional scalar factors from on-site steps. The SU(2) part of each contribution is entirely contained in U_p.
Equation (S120) makes explicit that the unitary dynamics organizes as a coherent superposition of contributions from all lattice paths, each carrying a path-dependent SU(2) spin rotation.

A. Spin observables, interference, and Wilson loops

a. Local spin observable. We consider the local spin operator at site j,

$$\hat{S}_{\alpha,j} \equiv |j\rangle\langle j| \otimes S_\alpha, \qquad S_\alpha = \frac{\hbar}{2}\sigma_\alpha, \tag{S121}$$

with $\alpha \in \{x, y, z\}$ and σ_α the Pauli matrices.
For an initial state localized on site i with spin $|\chi\rangle$,

$$|\Psi(0)\rangle = |i\rangle \otimes |\chi\rangle, \tag{S122}$$

the expectation value at time t is

$$\langle \hat{S}_{\alpha,j}(t)\rangle = \langle \Psi(0)|\hat{U}^\dagger(t)\, \hat{S}_{\alpha,j}\, \hat{U}(t)|\Psi(0)\rangle. \tag{S123}$$

Using the site projector in Eq. (S121) and the definition $\mathcal{U}_{ji}(t) = \langle j|\hat{U}(t)|i\rangle$, one obtains

$$\langle \hat{S}_{\alpha,j}(t)\rangle = \langle \chi|\, \mathcal{U}^\dagger_{ji}(t)\, S_\alpha \mathcal{U}_{ji}(t)\, |\chi\rangle. \tag{S124}$$

b. Reduction to the site-to-site spin propagator. Here we show explicitly how Eq. (S124) follows from Eq. (S123) for an initial state localized on site i. We take

$$|\Psi(0)\rangle = |i\rangle \otimes |\chi\rangle, \tag{S125}$$

and we recall the local spin operator at site j,

$$\hat{S}_{\alpha,j} = |j\rangle\langle j| \otimes S_\alpha. \tag{S126}$$

Starting from Eq. (S123) and substituting Eqs. (S125) and (S126), we obtain

$$\langle \hat{S}_{\alpha,j}(t)\rangle = (\langle i| \otimes \langle\chi|)\, \hat{U}^\dagger(t)\, (|j\rangle\langle j| \otimes S_\alpha)\, \hat{U}(t)\, (|i\rangle \otimes |\chi\rangle). \tag{S127}$$

Contracting first the site degrees of freedom yields

$$\langle \hat{S}_{\alpha,j}(t)\rangle = \langle\chi| \left(\langle i|\hat{U}^\dagger(t)|j\rangle\right) S_\alpha \left(\langle j|\hat{U}(t)|i\rangle\right) |\chi\rangle. \tag{S128}$$

Defining the spin-space propagator block $\mathcal{U}_{ji}(t) \equiv \langle j|\hat{U}(t)|i\rangle$ and using $\langle i|\hat{U}^\dagger(t)|j\rangle = \left(\langle j|\hat{U}(t)|i\rangle\right)^\dagger = \mathcal{U}^\dagger_{ji}(t)$, we arrive at

$$\langle \hat{S}_{\alpha,j}(t)\rangle = \langle\chi| \mathcal{U}^\dagger_{ji}(t)\, S_\alpha \mathcal{U}_{ji}(t)\, |\chi\rangle, \tag{S129}$$

which is Eq. (S124). Importantly, no commutation between $\mathcal{U}_{ji}(t)$ and S_α is assumed; S_α remains sandwiched between the two propagators.

c. Interference from the path expansion. Substituting the coherent path decomposition of the propagator,

$$\mathcal{U}_{ji}(t) = \sum_{p:i\to j} A_p(t)\, U_p, \qquad \mathcal{U}^\dagger_{ji}(t) = \sum_{p':i\to j} A^*_{p'}(t)\, U^\dagger_{p'}, \tag{S130}$$

into Eq. (S124) yields

$$\langle \hat{S}_{\alpha,j}(t)\rangle = \sum_{p,p':\, i\to j} A^*_{p'}(t)\, A_p(t)\, \langle\chi|\, U^\dagger_{p'}\, S_\alpha\, U_p\, |\chi\rangle. \tag{S131}$$

The terms with $p = p'$ are diagonal contributions, while the terms with $p \neq p'$ are cross terms encoding quantum interference between distinct paths connecting the same endpoints.

d. Emergence of Wilson loops. For any pair of paths (p, p') from i to j, we may insert the identity $U_p U_p^\dagger = \mathbf{1}$ to isolate the relative SU(2) rotation,

$$U^\dagger_{p'}\, S_\alpha\, U_p = \underbrace{(U^\dagger_{p'} U_p)}_{W(C)} \qquad \underbrace{(U^\dagger_p S_\alpha U_p)}_{\text{spin operator in the path-}p\text{ frame}} \qquad . \tag{S132}$$

The operator

$$W(C) \equiv U^\dagger_{p'} U_p \in SU(2) \tag{S133}$$

is the Wilson loop associated with the closed contour

$$C \equiv p \circ (p')^{-1}, \tag{S134}$$

i.e. the path obtained by traversing p from i to j and then returning from j to i along p' in reverse order. Since U_p and $U_{p'}$ are ordered products of link matrices U_{ji}, Eq. (S133) corresponds to the ordered SU(2) product around the closed loop C.

Equation (S131) together with Eqs. (S132)–(S133) shows that the SU(2) structure controlling path interference is governed by Wilson loops: whenever two distinct paths connect the same endpoints, their interference involves the relative spin rotation encoded in $W(C)$.

e. Time dependence and "independence" of the interference structure. Importantly, the time dependence in Eq. (S131) enters exclusively through the scalar weights $A_p(t)$, while the relative SU(2) rotation between two paths is entirely contained in $W(C) = U_{p'}^{\dagger} U_p$, which is fixed by the Hamiltonian through the link matrices. Thus, time controls the *weights* with which different paths contribute, whereas the *spin-dependent interference structure*, i.e. whether a nontrivial relative SU(2) phase exists between alternative paths, is determined solely by the Wilson loops.

f. Connection to steady-state transport (Green's functions). The same path-interference structure appears in the energy domain. The retarded Green's function admits the representation

$$G^R(E) = \frac{1}{E + i0^+ - \hat{H}} = -i \int_0^\infty dt\, e^{i(E+i0^+)t}\, \hat{U}(t), \tag{S135}$$

so that each matrix element $G^R_{ji}(E) = \langle j | G^R(E) | i \rangle$ is the (Laplace/Fourier) transform of $\mathcal{U}_{ji}(t)$. Inserting Eq. (S120) into Eq. (S135) shows that $G^R_{ji}(E)$ can be written as a coherent sum over the *same* path SU(2) factors U_p, with energy-dependent scalar weights given by the transform of $A_p(t)$. Consequently, the Wilson-loop factors governing spin-dependent interference are identical in real-time unitary dynamics and in steady-state transport formulations based on Green's functions.

g. Trivial Wilson loops, path independence, and vanishing polarization. If all Wilson loops are trivial, $W(C) = \mathbf{1}$ for any closed contour C. Then for any two paths p, p' connecting the same sites i and j one has $U_{p'}^{\dagger} U_p = \mathbf{1}$, implying $U_p = U_{p'}$. Therefore the SU(2) factor becomes path independent, and Eq. (S120) factorizes as

$$\mathcal{U}_{ji}(t) = A_{ji}(t)\, U^{(0)}_{ji}, \tag{S136}$$

where $U_{ji}^{(0)} \in SU(2)$ is a fixed spin rotation and $A_{ji}(t) = \sum_{p:i\to j} A_p(t)$ collects all scalar path amplitudes.

Substituting Eq. (S136) into Eq. (S124) yields

$$\langle \hat{S}_{\alpha,j}(t)\rangle = |A_{ji}(t)|^2 \, \langle\chi| \, (U_{ji}^{(0)})^\dagger S_\alpha U_{ji}^{(0)} \, |\chi\rangle, \tag{S137}$$

showing that the effect of propagation reduces to a single global SU(2) rotation of the initial spin state.

For an unpolarized initial state, described by the spin density matrix $\rho_{\text{spin}} = \mathbf{1}/2$, Eq. (S137) generalizes to

$$\langle \hat{S}_{\alpha,j}(t)\rangle = \frac{|A_{ji}(t)|^2}{2} \, \mathrm{Tr}\Big[(U_{ji}^{(0)})^\dagger S_\alpha U_{ji}^{(0)}\Big] = \frac{|A_{ji}(t)|^2}{2} \, \mathrm{Tr}[S_\alpha] = 0, \tag{S138}$$

since $\mathrm{Tr}[S_\alpha] = 0$. Thus, when all Wilson loops are trivial, spin polarization vanishes identically for unpolarized injection.

VIII. NUMERICAL VERIFICATION FOR COHERENT EVOLUTION

The initial state is constructed as a tensor product of an electronic state and a bosonic state:

- **Electronic part:** A density matrix is defined to have an occupation of 0.5 on the site 1 (both spins) and zero on the other sites: ρ_e
- **Bosonic part:** The bosonic density matrix represents the vacuum state: ρ_b

The total initial density matrix is then given by

$$\rho_0 = \rho_e \otimes \rho_b \, . \tag{S139}$$

The code defines a matrix $\mathbf{P}_{N,z}$ which, in the basis $[1\uparrow, 1\downarrow, 2\uparrow, 2\downarrow, ..., N\uparrow, N\downarrow]$, is zero for the first $N-1$ components and has σ_z for the N-th site, to calculate the polarization along z. Similarly, a matrix $\mathbf{P}_{N,x}$ is constructed that has σ_x in the N-th entry and a $\mathbf{P}_{N,y}$ with σ_y in the N-th entry to calculate the polarizations along x and y, respectively. This operator measures the difference in population or the spin polarization at the N-th site. Extending $\mathbf{P}_{N,i}$ to the full Hilbert space gives

$$\mathbf{P}_{N,i,full} = \mathbf{P}_{N,i} \otimes \mathbf{1}_{bos} \qquad i = x, y, z \, . \tag{S140}$$

Time Dynamics and Polarization Evolution:

The code defines a time interval and, for each time t, calculates the time evolution operator. The coherent time evolution was evaluated in the interval $t \in [0, 1]$ ps, with a uniform time step small enough to ensure numerical convergence of the exponential operator

$$\mathbf{U}(t) = e^{-i\frac{\mathbf{H}}{\hbar}t}\,, \tag{S141}$$

which is then used to update the density matrix

$$\rho(t) = \mathbf{U}(t)\rho_0\mathbf{U}^\dagger(t)\,. \tag{S142}$$

At each time step, the expectation value of the transformed polarization operator is computed as

$$P_{N,i}(t) = \mathrm{Re}\{\mathrm{Tr}(\rho(t)\cdot\hat{\mathbf{P}}_{N,i,full})\} \qquad i = x, y, z\,. \tag{S143}$$

Simulations:

For the simulations of figure S2 and S3 and the one of the main, a chain with $N = 12$ and a number of bosons $N_b = 50$ with $\hbar\omega_0 = 0.1\ eV$ was used. All parameters were randomly extracted from a Gaussian distribution using the Matlab command $rand(1)$. For example, for on-site energies, a value of $0.1\ eV$ was chosen and this was multiplied by $rand(1)$ on each site to obtain a different value. The same was done for the other couplings. We now report the starting value and then the vectors used for the reproducibility of the simulations. This was done in order to avoid introducing accidental symmetries into the Hamiltonian.

$$\epsilon_j = (0.1\ eV)\cdot rand(1) \qquad t_j = (0.2\ eV)\cdot rand(1) \qquad \lambda_j = (0.005\ eV)\cdot rand(1)$$

$$g_j^{Holstein} = (0.05\,eV)\cdot rand(1) \qquad g_j^{Peierls} = (0.05\,eV)\cdot rand(1) \qquad \chi_j = (0.005\,eV)\cdot rand(1)$$

$$v_{x,j} = 2\cdot rand(1) \qquad v_{y,j} = 2\cdot rand(1) \qquad v_{z,j} = 2\cdot rand(1)$$

The vector $\vec{v}$ was chosen to weight the SOC components differently without any geometric structure.

The explicit parameter values used in the simulations are reported below to ensure full reproducibility of the results.

$$\epsilon = [0.0939, 0.0876, 0.0550, 0.0622, 0.0587, 0.0208, 0.0301, 0.0471, 0.0230, 0.0844, 0.0195, 0.0226]$$

$$\mathbf{t} = [0.0516, 0.0817, 0.1190, 0.0524, 0.1206, 0.1422, 0.0443, 0.0235, 0.0593, 0.0638, 0.0848]$$

$$\lambda = [0.0025, 0.0004, 0.0013, 0.0040, 0.0001, 0.0046, 0.0037, 0.0024, 0.0029, 0.0012, 0.0023]$$

$$\mathbf{g}^{Holstein} = [0.0479, 0.0120, 0.0338, 0.0145, 0.0336, 0.0348, 0.0034, 0.0127, 0.0112, 0.0334, 0.0422, 0.0172]$$

$$\mathbf{g}^{Peierls} = [0.0265, 0.0327, 0.0204, 0.0410, 0.0359, 0.0484, 0.0266, 0.0163, 0.0053, 0.0305, 0.0389]$$

$$\chi = [0.0039, 0.0034, 0.0000, 0.0030, 0.0019, 0.0046, 0.0000, 0.0023, 0.0021, 0.0023, 0.0039]$$

$$\mathbf{v}_x = [0.6449, 1.5695, 0.9427, 0.0715, 0.3517, 1.4435, 0.9470, 0.3054, 0.6822, 1.2148, 0.3835]$$

$$\mathbf{v}_y = [1.4769, 0.4857, 1.8348, 0.5381, 1.5310, 0.3773, 0.5750, 0.1822, 1.1524, 1.3667, 1.0932]$$

$$\mathbf{v}_z = [0.8515, 1.2889, 1.2952, 1.3580, 1.2716, 1.8903, 0.4179, 1.4186, 0.4725, 0.2388, 1.2146]$$

Results along $\hat{z}$ and $\hat{y}$:

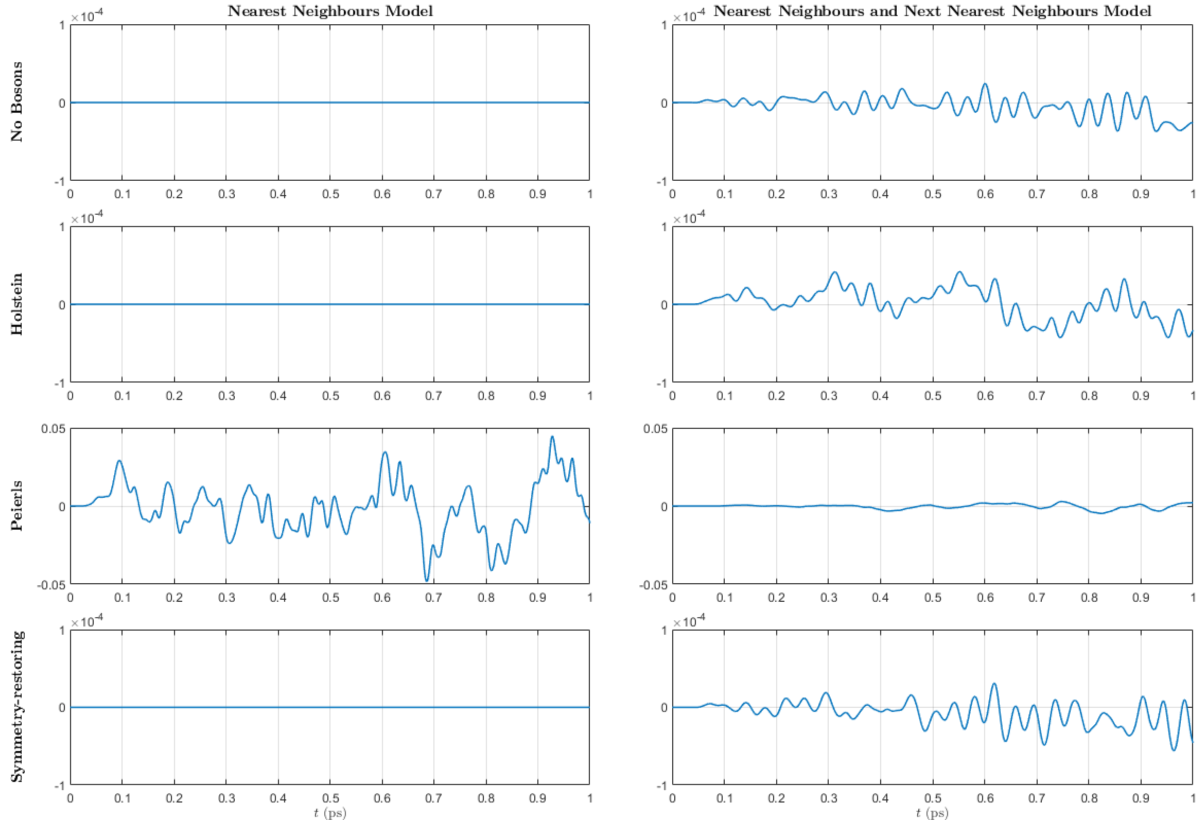

Figure S2: Time evolution of the spin polarization along the z axis at the site N, $\langle S_{z,N}(t)\rangle$, for different coupling schemes. The left column corresponds to the single-channel model (nearest-neighbour, NN), while the right column shows the multi-channel case including next-nearest-neighbour (NN+NNN) interactions. From top to bottom: (**i**) no bosonic coupling, (**ii**) Holstein-type electron–boson coupling, (**iii**) Peierls-type coupling, and (**iv**) the symmetry-restoring configuration. The vertical scale is the same for the first, second, and fourth rows, whereas the third row (Peierls case) uses a larger range ($[-0.05, 0.05]$) for visual clarity.

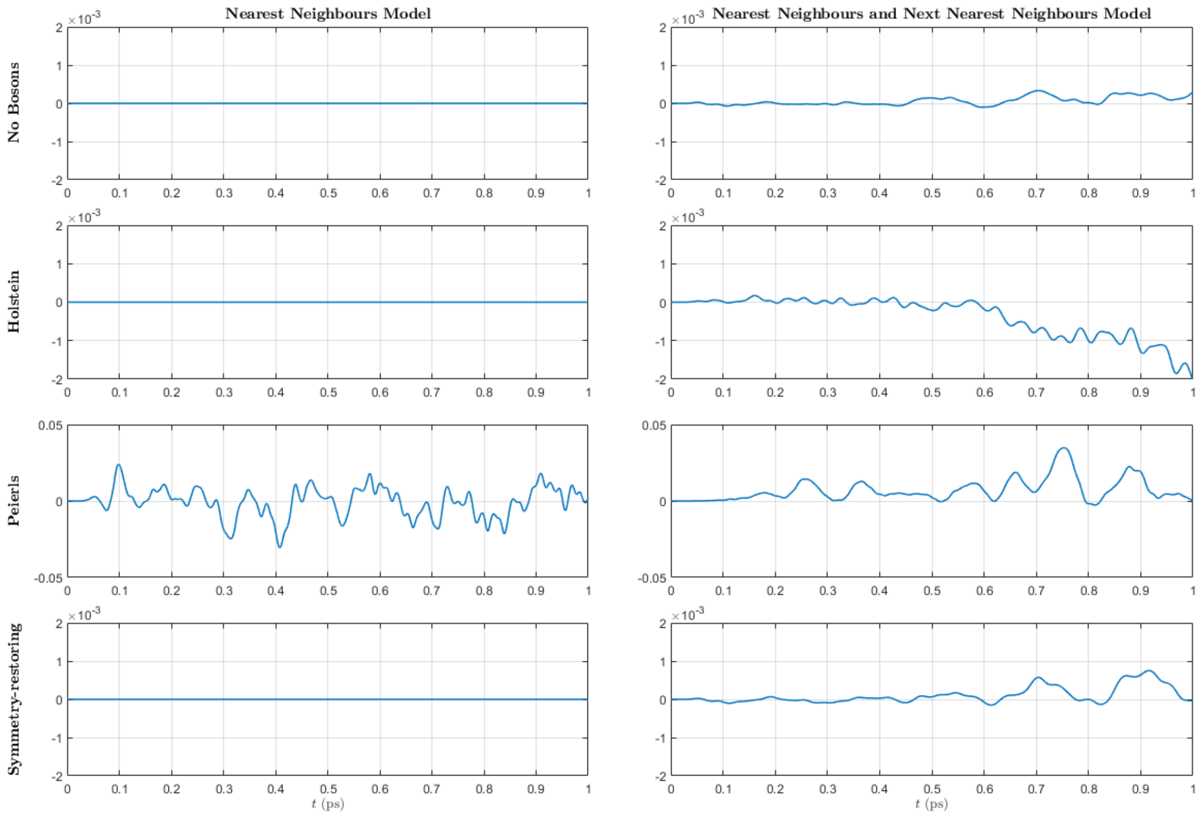

Figure S3: Time evolution of the spin polarization along the y axis at the site N, $\langle S_{y,N}(t)\rangle$, for different coupling schemes. The left column corresponds to the single-channel model (nearest-neighbour, NN), while the right column shows the multi-channel case including next-nearest-neighbour (NN+NNN) interactions. From top to bottom: (**i**) no bosonic coupling, (**ii**) Holstein-type electron–boson coupling, (**iii**) Peierls-type coupling, and (**iv**) the symmetry-restoring configuration. The vertical scale is the same for the first, second, and fourth rows, whereas the third row (Peierls case) uses a larger range ($[-0.05, 0.05]$) for visual clarity.

A. Wilson Loop

For the simulations of the chain with PBC, a closed chain with the same parameters seen above, with the addition of the hopping and SOC parameters between site N and site 1, extracted using the same criteria and reported here

$$t_{PBC} = 0.1812\ eV \qquad \lambda_{PBC} = 0.0046\ eV$$

$$v_{x,PBC} = 0.1951 \qquad v_{y,PBC} = 0.5570 \qquad v_{z,PBC} = 1.0938$$

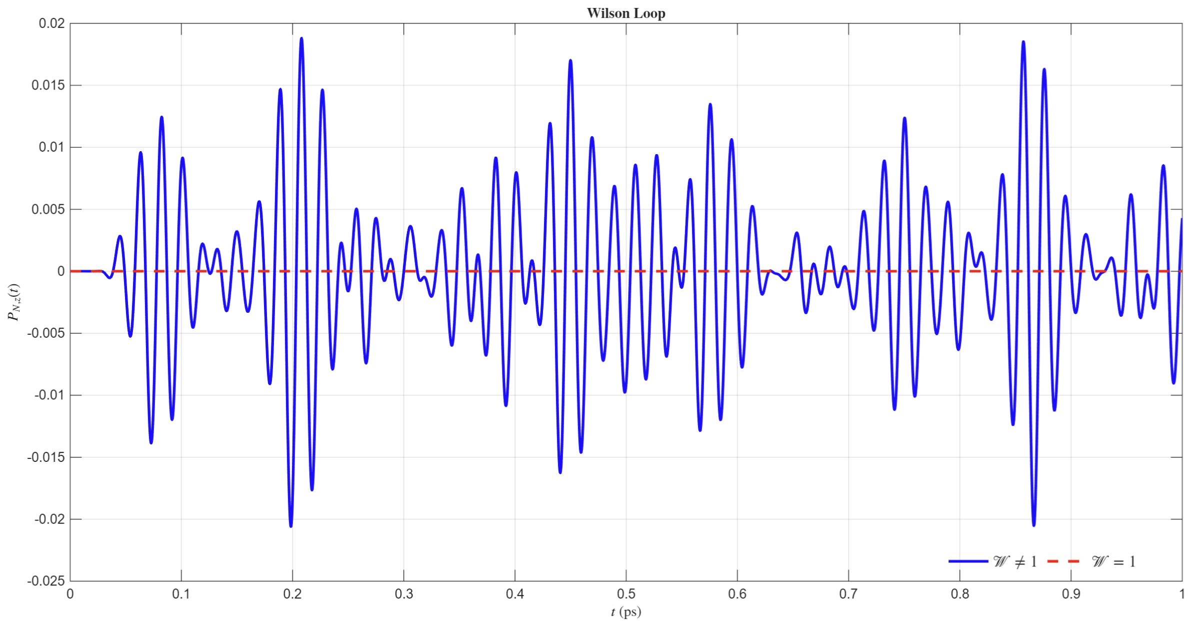

Figure S4: Numerical evaluation of $P_{z,N}(t)$ for the WL $\mathcal{W}$ for the model with periodic boundary conditions (PBC). The two curves correspond to the regimes $\mathcal{W} \neq \mathbf{1}$ (symmetry-broken phase) and $\mathcal{W} = \mathbf{1}$ (symmetry-restored phase), in agreement with the analytical condition discussed in Eq. (S18). The simulation confirms that the transition between these two regimes coincides with the restoration of spin symmetry observed in the polarization dynamics (see Fig. S2 and Fig. S3).

B. Color maps

For the simulations of the color maps, we considered a chain with $N = 6$ sites and a bosonic Hilbert space truncated at $N_b = 50$. The results are shown for phonon frequencies $\hbar\omega_0 = 0.05,\ 0.06,\ 0.08,$ and 0.10 eV. Here we used degenerate energies, hopping to first neighbors and SOC to second neighbors.

$$\epsilon_j = 0\ eV \qquad t_j = 0.1\ eV \qquad \lambda_j = 0.001\ eV \qquad g_j^{Holstein} = 0\ eV \qquad \forall j$$

Of the vector $\vec{v}$, we kept only the z component equal to 1 and measured the polarization along z for each of the 6 sites. For the color-map scans in (g, χ) we work in a fixed bosonic truncation with $N_b = 50$ levels and split the fermionic Hilbert space into particle-number sectors. With $N = 6$ sites and spin-$\frac{1}{2}$ ($N_{\text{orb}} = 12$ fermionic modes), we define

$$\mathcal{H}_{N_e=0} \equiv \{\, |\psi\rangle : N_{\text{tot}}\,|\psi\rangle = 0 \,\}, \qquad \mathcal{H}_{N_e=1} \equiv \{\, |\psi\rangle : N_{\text{tot}}\,|\psi\rangle = 1 \,\}, \qquad \text{(S144)}$$

where $N_{\rm tot}$ is the total electron-number operator. Correspondingly, we build the total Hamiltonian (electrons + boson) in the two sectors,

$$H^{(0)} = H_{\rm el}^{(0)} \otimes \mathbf{1}_{\rm bos} - \frac{g}{\sqrt{2}}\, H_{\rm hop}^{(0)} \otimes (a^\dagger + a) + \frac{\chi}{\sqrt{2}}\, H_{\rm SOC}^{(0)} \otimes (a^\dagger + a) + \mathbf{1} \otimes H_{\rm vib}, \tag{S145}$$

$$H^{(1)} = H_{\rm el}^{(1)} \otimes \mathbf{1}_{\rm bos} - \frac{g}{\sqrt{2}}\, H_{\rm hop}^{(1)} \otimes (a^\dagger + a) + \frac{\chi}{\sqrt{2}}\, H_{\rm SOC}^{(1)} \otimes (a^\dagger + a) + \mathbf{1} \otimes H_{\rm vib}, \tag{S146}$$

with $H_{\rm vib} = \hbar\omega_0\big(a^\dagger a + \frac{1}{2}\big)$ and $a^\dagger, a$ the bosonic ladder operators on a $(N_b{+}1) \times (N_b{+}1)$ space. We first diagonalize $H^{(0)}$ and take its ground state $|\Psi_0^{(0)}\rangle$ to form the pure density matrix

$$\rho_{00} = |\Psi_0^{(0)}\rangle\langle\Psi_0^{(0)}| \in \mathcal{H}_{N_e=0} \otimes \mathcal{H}_{\rm bos}. \tag{S147}$$

We then "inject" one electron on site 1 in an unpolarized way by acting with the spin-up and spin-down creation operators on site 1 and projecting onto the $N_e{=}1$ sector. Denoting $c_{1\uparrow}^\dagger$ and $c_{1\downarrow}^\dagger$ the fermionic creators reduced to the $\mathcal{H}_{N_e=0} \to \mathcal{H}_{N_e=1}$ block, the (unnormalized) state in the $N_e{=}1$ sector is

$$\tilde{\rho}_0 \;=\; (c_{1\uparrow}^\dagger \otimes \mathbf{1}_{\rm bos})\, \rho_{00}\, (c_{1\uparrow} \otimes \mathbf{1}_{\rm bos}) \;+\; (c_{1\downarrow}^\dagger \otimes \mathbf{1}_{\rm bos})\, \rho_{00}\, (c_{1\downarrow} \otimes \mathbf{1}_{\rm bos}). \tag{S148}$$

We finally normalize and express the state in the eigenbasis of $H^{(1)}$. Writing $H^{(1)} = V\, {\rm diag}(E_\alpha)\, V^\dagger$, the initial density matrix used in time evolution is

$$\rho_0 \;=\; \frac{V^\dagger\, \tilde{\rho}_0\, V}{{\rm Tr}\big(V^\dagger \tilde{\rho}_0 V\big)} \;\in\; \mathbb{C}^{D\times D}, \tag{S149}$$

with $D = \dim(\mathcal{H}_{N_e=1}) \times (N_b{+}1)$.

For the color maps we report the peak polarization at each site,

$$P_{z,k}^{\max}(g,\chi) \;=\; 2 \max_{t\in[0,\,1]\mathrm{ps}} |\langle S_{z,k}(t)\rangle| \;. \tag{S150}$$

The results are in Fig. S5, Fig. S6 and Fig. S7.

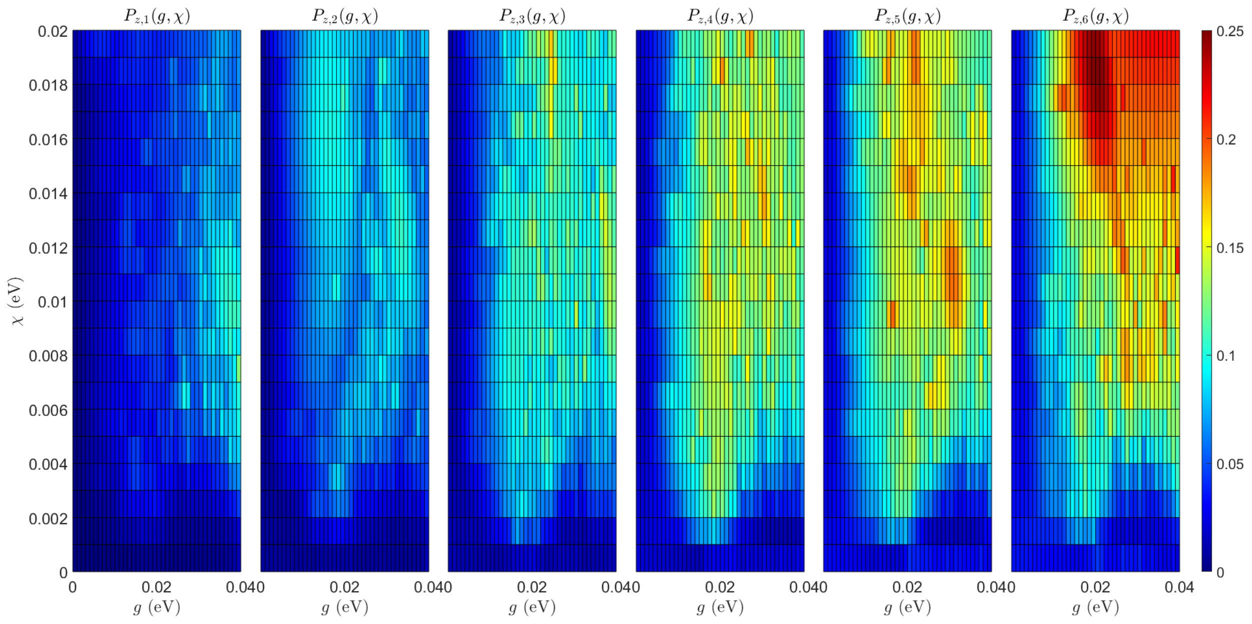

Figure S5: Same as Fig.5 of the main but for $\hbar\omega_0 = 60\,\mathrm{meV}$. The overall structure of the maps is unchanged.

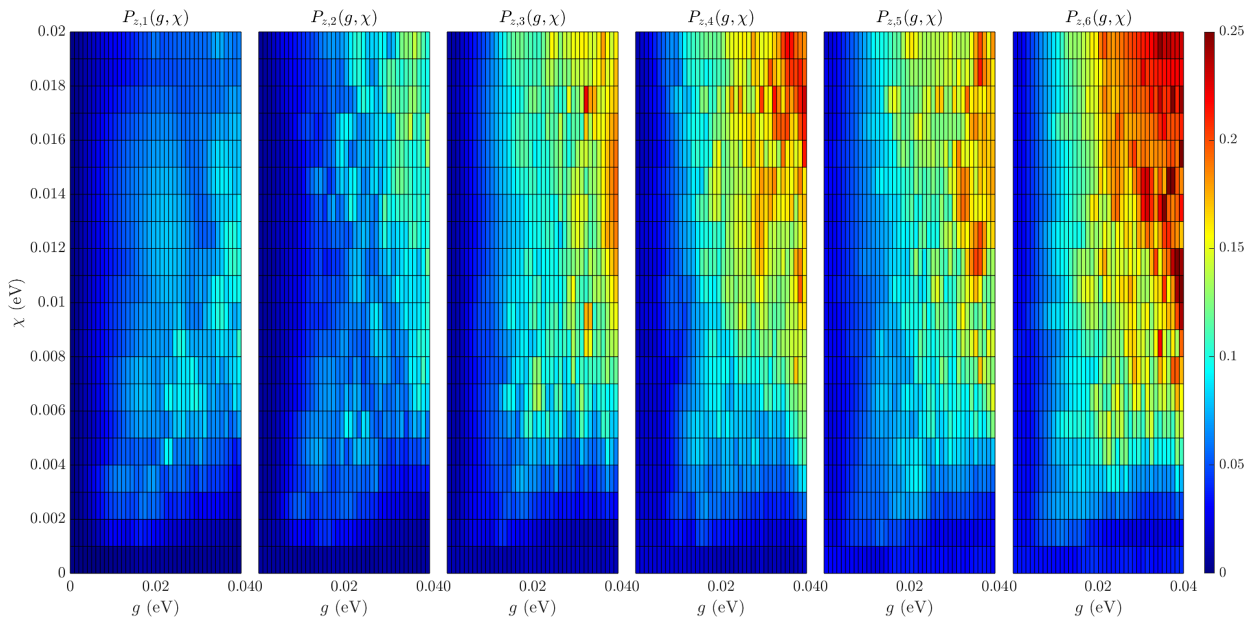

Figure S6: Same as Fig.5 of the main but for $\hbar\omega_0 = 80\,\mathrm{meV}$. The overall structure of the maps is unchanged.

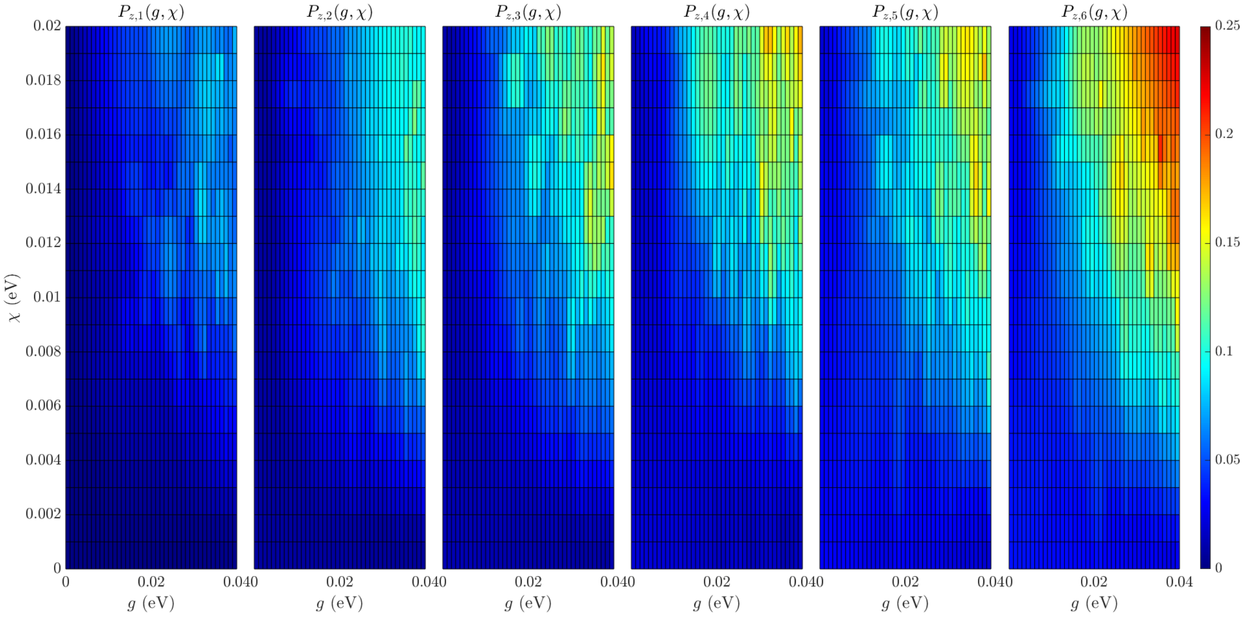

Figure S7: Same as Fig.5 of the main but for $\hbar\omega_0 = 100\,\text{meV}$. The overall structure of the maps is unchanged.

---

[1] J. Fransson, Chirality-induced spin selectivity: The role of electron correlations, J. Phys. Chem. Lett. **10**, 7126 (2019).

[2] N. Hatano, R. b. o. Shirasaki, and H. Nakamura, Non-abelian gauge field theory of the spin-orbit interaction and a perfect spin filter, Phys. Rev. A **75**, 032107 (2007).

[3] Q.-f. Sun, X. C. Xie, and J. Wang, Persistent spin current in nanodevices and definition of the spin current, Phys. Rev. B **77**, 035327 (2008).
